# Insights into the genetic diversity of an underutilized Indian legume, *Vigna stipulacea* (Lam.) Kuntz., using morphological traits and microsatellite markers

**DOI:** 10.1371/journal.pone.0262634

**Published:** 2022-01-19

**Authors:** Padmavati G. Gore, Veena Gupta, Rakesh Singh, Kuldeep Tripathi, Ramesh Kumar, Gita Kumari, Latha Madhavan, Harsh Kumar Dikshit, Kamala Venkateswaran, Anjula Pandey, Neeta Singh, Kangila V. Bhat, Ramakrishnan M. Nair, Aditya Pratap

**Affiliations:** 1 Division of Plant Genetic Resources, ICAR - Indian Agricultural Research Institute, New Delhi, India; 2 ICAR-National Bureau of Plant Genetic Resources, New Delhi, India; 3 ICAR-Indian Institute of Pulses Research, Kanpur, Uttar Pradesh, India; 4 ICAR-National Bureau of Plant Genetic Resources, Thrissur, Kerala, India; 5 Division of Genetics, ICAR-Indian Agricultural Research Institute, New Delhi, India; 6 ICAR-National Bureau of Plant Genetic Resources, Hyderabad, Telangana, India; 7 World Vegetable Center, South and Central Asia, Hyderabad, Telangana, India; National Institute for Plant Genome Research, INDIA

## Abstract

*Vigna stipulacea* (Lam.) Kuntz., commonly known as *Minni payaru* is an underutilized legume species and has a great potential to be utilized as food crop. To evaluate and select the best germplasm to be harnessed in the breeding programme, we assessed the genetic diversity of *V*. *stipulacea* (94 accessions) conserved in the Indian National Genebank, based on morphological traits and microsatellite markers. Significant variation was recorded for the morphological traits studied. Euclidean distance using UPGMA method grouped all accessions into two major clusters. Accessions were identified for key agronomic traits such as, early flowering (IC331436, IC251436, IC331437); long peduncle length (IC553518, IC550531, IC553557, IC553540, IC550532, IC553564); and more number of seeds per pod (IC553529, IC622865, IC622867, IC553528). To analyse the genetic diversity among the germplasm 33 SSR primers were used anda total of 116 alleles were detected. The number of alleles varied from two to seven, with an average of 3.52 per loci. The polymorphic information content values varied from 0.20 to 0.74, with a mean of 0.40. The high number of alleles per locus and the allelic diversity in the studied germplasm indicated a relatively wider genetic base of *V*. *stipulacea*. Phylogenetic analysis clustered accessions into seven clades. Population structure analysis grouped them into five genetic groups, which were partly supported by PCoA and phylogenetic tree. Besides, PCoA and AMOVA also decoded high genetic diversity among the *V*. *stipulacea* accessions. Thus, morphological and microsatellite markers distinguished *V*. *stipulacea* accessions and assessed their genetic diversity efficiently. The identified promising accessions can be utilized in *Vigna* improvement programme through introgression breeding and/or can be used for domestication and enhanced utilization of *V*. *stipulacea*.

## Introduction

The *Vigna* is an important genus among all pulse crops, containing more than 200 domesticated and wild species [1]. Among these, the domesticated species, namely, green gram or mungbean [*V*. *radiata* (L.) Wilczek], black gram or urdbean [*V*. *mungo* (L.) Hepper], cowpea or *lobia* [*V*. *unguiculata* (L.)Walp], adzuki bean [*V*. *angularis* (Willd.) Ohwi and Ohashi], bambara groundnut [*V*. *subterranea* (L.) Verdn.], moth bean [*V*. *aconitifolia* (Jacq.)] and rice bean [*V*. *umbellata* (Thunb.) Ohwi and Ohashi] are of commercial importance and contribute significantly to the pulse production [1, 2]. While these crops have a positive impact on food and nutritional security worldwide and also on environmental sustainability, their productivity and production remain low owing to several biotic and abiotic constraints [3, 4]. Several *Vigna* species have the status of under-exploited, undomesticated or semi-domesticated species or simply ‘wild *Vignas’* [5–8]. Collection, evaluation and investigation of hitherto wild and exotic species from different agro-climatic regions to domesticate the undomesticated ones remains an important method, which could help mitigate the food and nutritional insecurity concerns [9].

Among various wild and useful *Vigna* species, *Vigna stipulacea* (Lam) Kuntz., is an important one with tremendous potential to be utilized in the *Vigna* improvement programme. The genome size of *V*. *stipulacea* is ~445.1 Mbp [10], and it belongs to the secondary gene pool of the cultivated green gram and black gram. This species is a reservoir of various useful traits *viz*., faster growth, shorter duration and broad resistance to various pests and diseases and can be targeted for its domestication as a new crop and while the desirable traits can be transferred to the cultivated *Vigna* species [10]. Though *V*. *stipulacea* has been mistaken with *V*. *trilobata* since a long, it is taxonomically distinct [11]. It is being grown traditionally in lowland paddy fields before paddy cultivation in South India and locally known as ‘*Minni payaru’* or “*Siru Payaru*” [12, 13]. This crop is primarily used for fodder and manure production [14, 15] and also being utilized as an ingredient for the preparation of vegetable stew (*sambhar*), condiment and *dosa* [12]. This species has been recognized as a source of disease and pest resistance [16–19]. Despite its economic importance, the information on the extent of variability on the *V*. *stipulacea* germplasm remains veiled. The National Gene Bank (NGB) of India housed at ICAR-National Bureau of Plant Genetic Resources (ICAR-NBPGR), New Delhi has conserved germplasm of *V*. *stipulacea*. This gene bank collection needed to be evaluated using the agro-morphological traits and efficient molecular markers like microsatellite markers (Simple Sequence Repeats) to assess the extent of diversity and identify promising germplasm lines. Information on the extent of genetic diversity of a panel of genotype and its population genetic structure provides useful information for deploying marker assisted breeding for genetic improvement [1, 20]. To use wild and underutilized species in crop improvement programmes through introgression breeding, precise information on their genetic architecture and population genetic structure and relationship with other *Vigna* species are a deciding factor [21]. The objectives of the present investigation were to evaluate the genetic diversity and study the population genetic structure of *V*. *stipulcaea* germplasm using agro-morphological and microsatellite markers which will provide a better insight into the extent of relatedness and diversity in the studied accessions. The identified germplasm as a result of the above efforts can be deployed for transferring traits of interest in cultivated background from the untapped wild genetic resources of *V*. *stipulacea*. This could help to shape the *Vigna* improvement progamme better and contribute significantly to ensuring global food and nutrition security.

## Materials and methods

### Plant materials

A diverse panel of 94 accessions (acc.) of *V*. *stipulacea* conserved in the NGB housed at ICAR-NBPGR, New Delhi was used in the present study. These accessions belonged to different diversity-rich agro-climatic regions of India and were collected from different states, including Andhra Pradesh (61 acc.), Tamil Nadu (14 acc.), Chhattisgarh (5 acc.), Madhya Pradesh (5 acc.), Odisha (3acc.) and Gujarat, Karnataka and Kerala (2 acc. each). The detailed passport data of all the accessions are presented in the Table 1.

**Table 1:** Details of passport data of *V*. *stipulacea* germplasm used in the study.

SN	Accession No	District	Indian State
**1**	IC252016	Kurnool	Andhra Pradesh
**2**	IC261321	Kurnool	Andhra Pradesh
**3**	IC261384	Kurnool	Andhra Pradesh
**4**	IC305192	Kurnool	Andhra Pradesh
**5**	IC553494	Kurnool	Andhra Pradesh
**6**	IC610275	Kurnool	Andhra Pradesh
**7**	IC524667	Cuddapah	Andhra Pradesh
**8**	IC550531	Vizianagaram	Andhra Pradesh
**9**	IC550532	Vizianagaram	Andhra Pradesh
**10**	IC550533	Vizianagaram	Andhra Pradesh
**11**	IC550536	Vizianagaram	Andhra Pradesh
**12**	IC550538	Vizianagaram	Andhra Pradesh
**13**	IC550545	Srikakulam	Andhra Pradesh
**14**	IC550548	Srikakulam	Andhra Pradesh
**15**	IC550551	Srikakulam	Andhra Pradesh
**16**	IC550553	Vishakhapatnam	Andhra Pradesh
**17**	IC553502	Mahbubnagar	Andhra Pradesh
**18**	IC524639	Prakasam	Andhra Pradesh
**19**	IC553505	Prakasam	Andhra Pradesh
**20**	IC553509	Prakasam	Andhra Pradesh
**21**	IC553510	Prakasam	Andhra Pradesh
**22**	IC553512	Prakasam	Andhra Pradesh
**23**	IC553516	Prakasam	Andhra Pradesh
**24**	IC553517	Prakasam	Andhra Pradesh
**25**	IC553518	Prakasam	Andhra Pradesh
**26**	IC553520	Prakasam	Andhra Pradesh
**27**	IC553521	Prakasam	Andhra Pradesh
**28**	IC553522	Prakasam	Andhra Pradesh
**29**	IC553523	Prakasam	Andhra Pradesh
**30**	IC553524	Prakasam	Andhra Pradesh
**31**	IC553525	Prakasam	Andhra Pradesh
**32**	IC553526	Prakasam	Andhra Pradesh
**33**	IC553534	Prakasam	Andhra Pradesh
**34**	IC553535	Prakasam	Andhra Pradesh
**35**	IC553527	Nellore	Andhra Pradesh
**36**	IC553528	Nellore	Andhra Pradesh
**37**	IC553529	Nellore	Andhra Pradesh
**38**	IC553530	Nellore	Andhra Pradesh
**39**	IC553531	Nellore	Andhra Pradesh
**40**	IC553532	Nellore	Andhra Pradesh
**41**	IC553537	Krishna	Andhra Pradesh
**42**	IC553538	Krishna	Andhra Pradesh
**43**	IC553539	Krishna	Andhra Pradesh
**44**	IC553540	Krishna	Andhra Pradesh
**45**	IC553541	Krishna	Andhra Pradesh
**46**	IC553544	Krishna	Andhra Pradesh
**47**	IC553547	Krishna	Andhra Pradesh
**48**	IC553548	Krishna	Andhra Pradesh
**49**	IC553551	Krishna	Andhra Pradesh
**50**	IC550520	West Godavari	Andhra Pradesh
**51**	IC553553	West Godavari	Andhra Pradesh
**52**	IC553554	West Godavari	Andhra Pradesh
**53**	IC553555	West Godavari	Andhra Pradesh
**54**	IC553556	West Godavari	Andhra Pradesh
**55**	IC553557	West Godavari	Andhra Pradesh
**56**	IC553558	West Godavari	Andhra Pradesh
**57**	IC553560	West Godavari	Andhra Pradesh
**58**	IC553561	West Godavari	Andhra Pradesh
**59**	IC553562	West Godavari	Andhra Pradesh
**60**	IC553564	West Godavari	Andhra Pradesh
**61**	IC553565	West Godavari	Andhra Pradesh
**62**	IC622860	Rewa	Madhya Pradesh
**63**	IC622861	Rewa	Madhya Pradesh
**64**	IC276983	Raisen	Madhya Pradesh
**65**	IC622865	Bhopal	Madhya Pradesh
**66**	NS_1	Bhopal	Madhya Pradesh
**67**	IC251435	Junagadh	Gujarat
**68**	BD_1	-	Gujarat
**69**	IC251436	-	Odisha
**70**	IC331436	Khurda	Odisha
**71**	IC331437	Ganjam	Odisha
**72**	IC331453	Raipur	Chhattisgarh
**73**	IC331454	Raipur	Chhattisgarh
**74**	IC331456	Bilaspur	Chhattisgarh
**75**	IC331457	Bilaspur	Chhattisgarh
**76**	IC331610	Bilaspur	Chhattisgarh
**77**	IC251438	Coimbatore	Tamil Nadu
**78**	IC349701	Coimbatore	Tamil Nadu
**79**	IC351406	Villupuram	Tamil Nadu
**80**	IC417392	Coimbatore	Tamil Nadu
**81**	IC622867	Trichy	Tamil Nadu
**82**	IC622868	Trichy	Tamil Nadu
**83**	IC622869	Trichy	Tamil Nadu
**84**	IC521211	Madurai	Tamil Nadu
**85**	IC521245	Trichy	Tamil Nadu
**86**	IC521215	Ramnathapuram	Tamil Nadu
**87**	PK_101	-	Tamil Nadu
**88**	IC037804	-	Tamil Nadu
**89**	IC622870	Trichy	Tamil Nadu
**90**	BDD_1	-	Tamil Nadu
**91**	IC421767	Belgaum	Karnataka
**92**	IC024830	-	Karnataka
**93**	NV_1	Idukki	Kerala
**94**	IC625694	Idukki	Kerala

All the accessions were grown at ICAR- Indian Institute of Pulses Research (ICAR-IIPR), Kanpur, Uttar Pradesh (26°27’N latitude, 80°14’E longitude, 452.4 meter above mean sea level (AMSL) for two consecutive years, i.e. during the rainy season (*Kharif)* 2018 and 2019. The long term mean annual rainfall of Kanpur is 820 mm, and >80% of it is received during the southwest monsoon season (July–September). All accessions were raised in customized cement pots using Augmented Block design (ABD)in the wide hybridization garden facility at ICAR-IIPR, Kanpur (S1 Fig) Each pot measured 1 m in diameter and was about 1 m high, and was filled with a potting mixture of sand: soil: FYM in 1:1:1 ratio. Further, each pot has a provision of drainage at the bottom and the sides so that water stagnation does not occur during excess precipitation. Before sowing, mechanical scarification of seeds was done in all accessions to ensure maximum germination [1, 22].

### Recording of data

The observations for 33 agro-morphological traits were recorded using descriptors of Bioversity International and ICAR-NBPGR [23].

The following 15 quantitative traits were recorded according to descriptors:

1. Stipule length (mm): measured in mm by taking 5 stipules for each accession.
2. Stipule width (mm): measured in mm by taking 5 stipules for each accession.
3. Terminal leaflet length (cm): recorded for the leaf at the fourth node and categorized as Small (<10 cm), Medium (10–13 cm), Large (>13 cm).
4. Petiole length (cm): recorded for the leaf at the fourth node and categorized as Short (<12 cm), Medium (12–18 cm), Long (>18 cm).
5. Peduncle length (cm): recorded for the length of the longest peduncle when the first pod changes colour and categorized as Short (<14 cm) 2), Medium (14–18 cm) 3), Long (>18 cm).
6. Branch length (cm): Measured in cm for the longest branch for 5 plants in each accession.
7. SPAD valueat 30 days: Measured using SPAD after 30 days of sowing
8. Plant height (cm): Five plants were selected randomly and plant height was measured from the base of plant at ground surface to the top in cm.
9. Days to initial flowering: Days in number from sowing date to 50% of plants with first flower open.
10. Days to first podding: Days in number from sowing date to 50% of plants with first pod development.
11. Days to initial maturity: Number of days from planting to 50% of plants with first ripe pod
12. Number of seeds per pod: recorded after harvesting by manually counting the seeds per pod and averaged for 10 pods per accession.
13. Pod length (cm): recorded for ten random pods and averaged in each accession in cm.
14. Seed length (mm): measured using Vernier calliper and expressed in mm.
15. Seed width (mm): measured using Vernier calliper and expressed in mm.

### Qualitative traits

Eighteen qualitative traits included, seed germination habit, hypocotyl colour, attachment of primary leaves, early plant vigour, stipule shape, leaf colour, branch pigmentation, petiole colour, leafiness, leaf pubescence, plant growth habit, attachment of pod to peduncle, pod pubescence, pod colour, constriction between seeds in the pod, seed shape, luster on seed surface and mottling on seed surface. Data on qualitative traits were recorded as per the descriptors and the details are presented in the S1 Table.

### Extraction of DNA

For DNA extraction Cetyl trimethylammonium bromide (CTAB) method was followed [24]. DNA quantification were completed with 1% agarose gel electrophoresis and confirmed by using spectrophotometer (Nanodrop^™^, Thermo-Fisher, USA) and final DNA concentration was adjusted to 20 ng/μL.

### SSR markers and Polymerase Chain Reaction (PCR) amplification

Initially, 100 microsatellite markers from different *Vigna* backgrounds viz., cowpea [25], adzuki bean [26], mungbean [27–31] and common bean [32, 33] (listed in supplementary data, S2 Table) were used to identify polymorphic markers on a panel of 20 accessions. Out of these, 33 SSR primers showed polymorphism, which were further, used for polymerase chain reaction (PCR) amplification in all the 94 accessions of *V*. *stipulacaea*. PCR reaction was set for 10 μl of total volume containing 10X buffer (1.25 μl), distilled water (1.8 μl), 25 mM MgCl2 (1.25 μl), 0.2 mM dNTPs mix (1.0 μl), 10 nmol Primer (F/R) (0.5 μl), Taq DNA polymerase (0.2 μl), 20 ng/μl DNA (4 μl). PCR conditions were set as suggested by Banger et al., (2018) [31] with minor modifications. The details of standardized program for amplification were as follows: initial denaturation was carried out for 6 min at 94°C, followed by 35 cycles of denaturation for 1 min at 94°C, annealing for 1 min at 48–52°C temperature (standardization of annealing temperature was carried out by gradient PCR for each SSR marker), extension for 1 min at 72°C and final extension for 10 min at 72°C. The amplified product was separated on 3% metaphor agarose (Lonza, USA) gel for 3h at 110 V and gel images were captured by using gel documentation system (Alpha Imager1, USA).

### Data scoring and statistical analyses

The data on morphological traits of both the years were pooled to calculate mean, standard error, standard deviation and coefficient of variation (%). These accessions were classified using hierarchical cluster analysis, Euclidean distances were estimated and a dendrogram was constructed following UPGMA tree method. PCA was used to assess the phenotypic diversity. Each principal component was computed by using a linear combination of an Eigen vector of the correlation matrix with a variable. The three-dimensional plot was also developed to depict the view of accession scores. Both hierarchical clustering and PCA were analyzed using NTSYS pc- 2.11x software [34].

Among the 94 *V*. *stipulacea* accessions, amplified products of 33 SSR loci were scored manually comparing with DNA ladder 100 bp (G-Biosciences, USA). The genetic diversity indices, viz., minor allele frequency (Maf), number of alleles (An), observed heterozygosity (Ho), genetic diversity (GD) or expected heterozygosity and polymorphism information content (PIC) of each SSR locus were calculated using PowerMarker v3.25 [35]. In addition, genetic distances across the *V*. *stipulacea* accessions and Neighbour-joining (NJ) tree were constructed using Power Marker v3.25 [35]. Population structure for *V*. *stipulacea* accessions was determined using the Bayesian model-based programme, software STRUCTURE 2.3.4 [36]. The number of clusters (K) was ranged from two to ten and three replications were run for analysis of each K value. The STRUCTURE was run with 100,000 generations of each burn-in and a Markov chain Monte Carlo. LnP(D) was obtained for each K and ΔK values are derived after plotting LnP(D) [37]. Final number of the populations of the studied accessions was obtained by using the STRUCTURE harvester. The data sets were subjected to Principle Coordinate Analysis (PCoA) and analysis for molecular variation (AMOVA) using software GenAlEx V6.5 [38].

## Results

### Quantitative traits exploration

**Fig 1:**
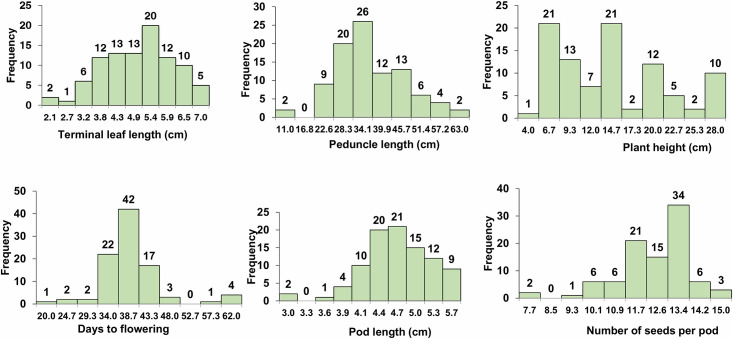
Frequency distribution of *V*. *stipulacea* accessions for quantitative traits.

Descriptive statistics of 15 quantitative traits indicated that significant variation was present among all accessions (Table 2, Fig 1, S3 Table, S2 Fig)). Among different morphological traits, the stipule length varied from 1.10 to 2.63 mm with a mean of 1.70 mm. Accession IC553553 collected from Andhra Pradesh recorded the highest stipule length. Accessions IC251435, IC331436, IC331437 (belonging to Odisha) and IC521245, IC37804 (Tamil Nadu) had lowest stipule length. In our study, a high coefficient of variation (30.22%) was observed for peduncle length. The longest peduncle length (63.0 cm) was recorded in the accession IC553564. Most of the accessions from Andhra Pradesh had the peduncle length more than the mean value (33.55 cm). Length of the longest branch showed significant variation in all the accessions ranging from 45 cm to 170 cm with a mean of 100 cm. The accessions IC251435, IC331436, IC331437 (belonging to Odisha) had branch length lower than the mean value while the accessions IC622860, IC622861, IC276893, IC622865 (Madhya Pradesh) had branch length closer to mean value. The maximum coefficient of variation was recorded for plant height (52.91%) with accession IC553532 recording highest plant height (28.0 cm) and IC349701 observes the lowest plant height (4.0 cm). The accession IC550533 had the longest terminal leaflet (7.03 cm), while accessions IC622870 recorded the minimum terminal leaflet length (2.13 cm). The soil plant analysis development (SPAD) value indicating the nitrogen status of the plants at 30 days ranged from 35.57 to 46.03, with an average value of 41.57. Days to initial flowering significantly varied among accessions. The mean value of 37 days was recorded for days to initial flowering. Minimum days to initial flowering were recorded for accessions *viz*., IC331436 (20 days), IC251436 (21 days) and IC331437 (22 days) which all belonged to Odisha while the maximum number of days to initial flowering were recorded for the accessions from Chhattisgarh *viz*., IC331610 (62 days), IC331453 (60 days), IC331457 (59 days), IC331456 (58 days) and IC331454 (56 days). Likewise, days to initial maturity also recorded significant variation and ranged between 49 to 76 days. Among the accessions studied, IC331436 matured at the earliest as it matured within 49 days after sowing. Other nine accessions namely, IC251436, IC331437, IC622861, IC276983, IC622865, IC622867, IC622860, IC331454, IC331457 matured in less than 60 days. A wide variability for pod length was observed. Pod length varied from 3 to 5.57 cm with an average of 4.57 cm. The accessions IC553516 and IC331453 had the minimum pod length (3 cm) whereas IC553509 (5.57 cm) had the maximum pod length. Seven to 15 numbers of seeds per pod with an average of 12 were recorded. Minimum number of seeds per pod was observed in the accessions IC610275 and IC553516 (7 each). The accessions IC553529, IC622865 and IC622867, recorded the highest number of seeds (15), followed by IC553528 (14). Seed length varied from a minimum of 1.76 mm to a maximum of 3.17 mm with an average of 2.64 mm. The accession IC037804 recorded highest seed length (3.17 mm) followed by IC622870 (3.13mm) while IC553548 observed lowest seed length of 1.76 mm. Likewise, seed width varied from a minimum of 1.47 mm to a maximum of 2.85 mm with average 2.12 mm. IC622867 (1.47 mm) recorded the lowest seed width, while IC553535 recorded the highest (2.85 mm).

**Table 2:** Descriptive statistics of quantitative characters of *V*. *stipulacea*.

S.N.	Traits	Min	Max	Mean±SE	SD	CV (%)
1.	Stipule length (mm)	1.10	2.63	1.70±0.04	0.42	24.65
2.	Stipule width (mm)	0.90	2.12	1.26±0.03	0.25	20.02
3.	Petiole length (cm)	3.50	17.67	10.21±0.32	3.08	30.19
4.	Peduncle length (cm)	11.00	63.00	33.55±1.05	10.14	30.22
5.	Branch length (cm)	45.00	170.00	100.11±2.75	26.64	26.61
6.	Plant height (cm)	4.00	28.00	13.47±0.74	7.13	**52.91**
7.	Terminal leaf length (cm)	2.13	7.03	4.74±0.12	1.14	24.12
8.	SPAD value at 30 days	35.57	46.03	41.57±0.29	2.82	**6.79**
9.	Days to initial flowering	20.00	62.00	37.24±0.69	6.72	18.05
10.	Days to first podding	23.00	66.00	41.00±0.69	6.74	16.43
11.	Days to initial maturity	49.00	76.00	65.44±0.52	5.06	**7.74**
12.	Pod length (cm)	3.00	5.57	4.57±0.06	0.53	11.68
13.	Seeds per pod	7.67	15.00	12.07±0.14	1.39	11.50
14.	Seed length (mm)	1.76	3.17	2.64±0.03	0.24	9.25
15.	Seed width (mm)	1.47	2.85	2.12±0.02	0.23	10.97

### Hierarchical clustering and PCA

**Fig 2:**
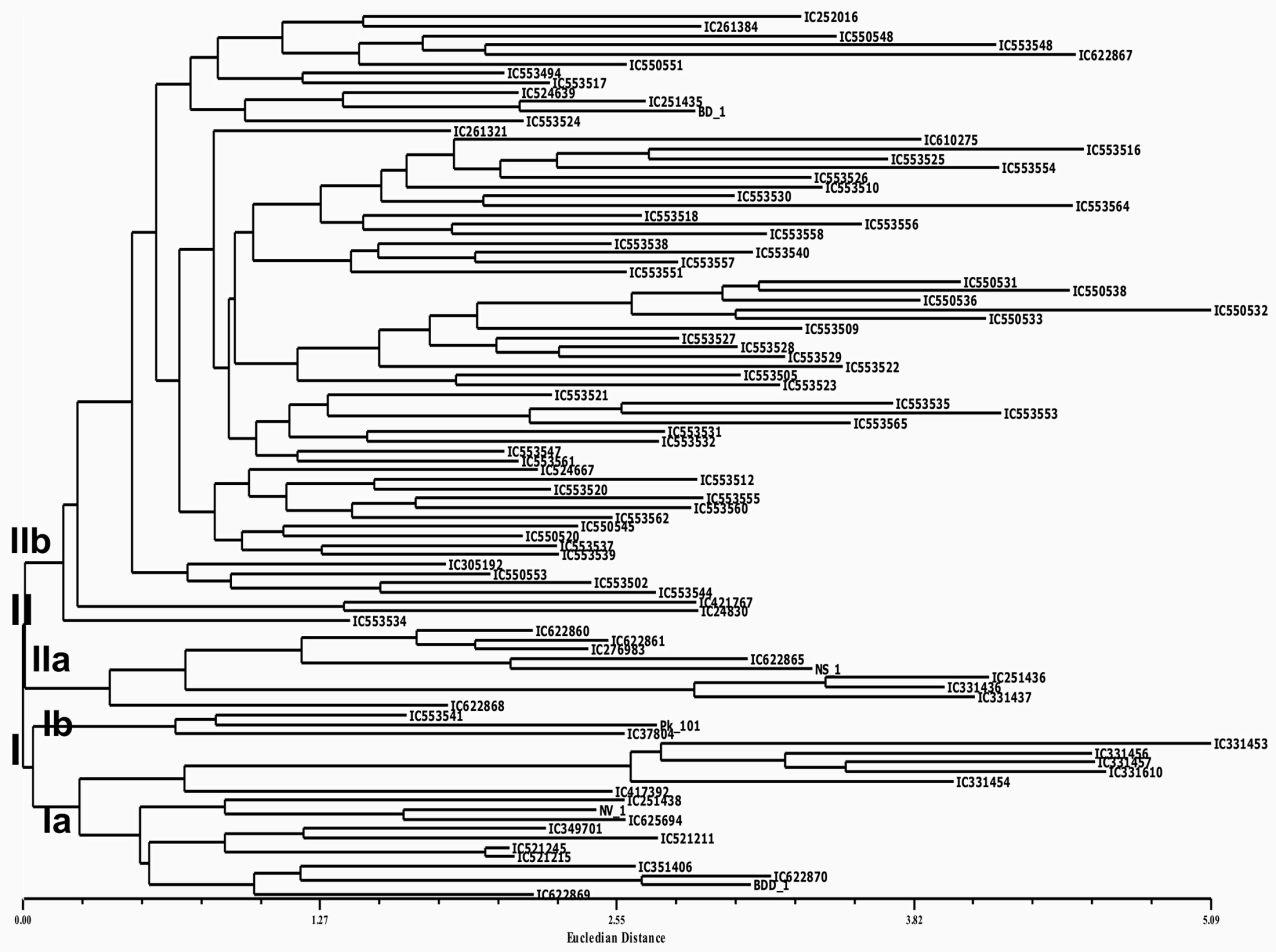
Dendrogram based on Euclidian distance using NJ tree method of clustering depicting genetic diversity and relationship between 94 accessions of *V*. *stipulacea* as revealed by quantitative traits.

Dendrogram based on the Euclidean distances grouped 94 accessions of *V*. *stipulacea* into two major clusters. Both the clusters were subsequently subdivided into two sub-clusters each, represented as Ia, Ib, IIa and IIb (Fig 2). The accessions belonging to Chhattisgarh were grouped into sub-cluster Ia whereas, accessions from Odisha were grouped in sub-cluster Ib, which comprised of early maturing accessions. Accessions in sub-clusters IIa and IIb did not group according to their geographical origin.

The first three principal components summarized a realistic summary of all data explaining 56.26% variance in 94 accessions of *V*. *stipulacea*. Most important was the first principal component (PC1) accounting for 27.18% of the total variation. PC2 and PC3 accounted for 17.68% and 11.40% variance, respectively. PC 1 explained variation in stipule length and terminal leaf length, PC2 in days to initial flowering, days to first podding and branch length while PC3 in pod length and number of seeds per pod (Table 3).

**Table 3:** Principal Component (PC) matrix explaining loading of variables.

Variables	PC1	PC2	PC3
Stipule length (mm)	.193	-.084	.029
Stipule width (mm)	.184	-.073	.153
Petiole length (cm)	.163	.186	.057
Peduncle length (cm)	.182	.056	.037
Branch length (cm)	.067	.219	-.003
Plant height (cm)	.187	-.105	.074
Terminal leaf length (cm)	.199	-.099	.020
SPAD value at 30 days	.003	.007	.098
Days to initial flowering	.026	.354	.044
Days to first podding	.037	.350	.025
Days to initial maturity	.129	-.035	.022
Pod length (cm)	-.031	-.067	.449
Number of seeds per pod	-.063	.023	.441
Seed length (mm)	.017	.024	-.272
Seed width (mm)	.103	-.051	-.260
**Percent variance**	**27.18**	**17.68**	**11.40**
**Cumulative variance (%)**	**27.18**	**44.86**	**56.26**

### Qualitative traits exploration

Qualitative traits were recorded at the appropriate stages of the plant growth when these had full expression. Accordingly, these traits were recorded at the germination, vegetative, podding and maturity stage as follows:

#### Germination stage

**Fig 3:**
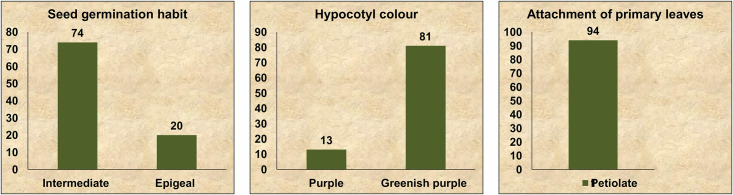
Variation in qualitative traits at germination stage among 94 accessions of *V*. *stipulacea*.

The seed germination habit, hypocotyl colour, attachment of leaf at the two-leaf stage (primary leaf) were observed at the time of germination. Most of the accessions showed an intermediate type of germination and greenish-purple coloured hypocotyls. Petiolate type of primary leaf attachment was observed in all accessions (Fig 3).

#### Vegetative stage

**Fig 4:**
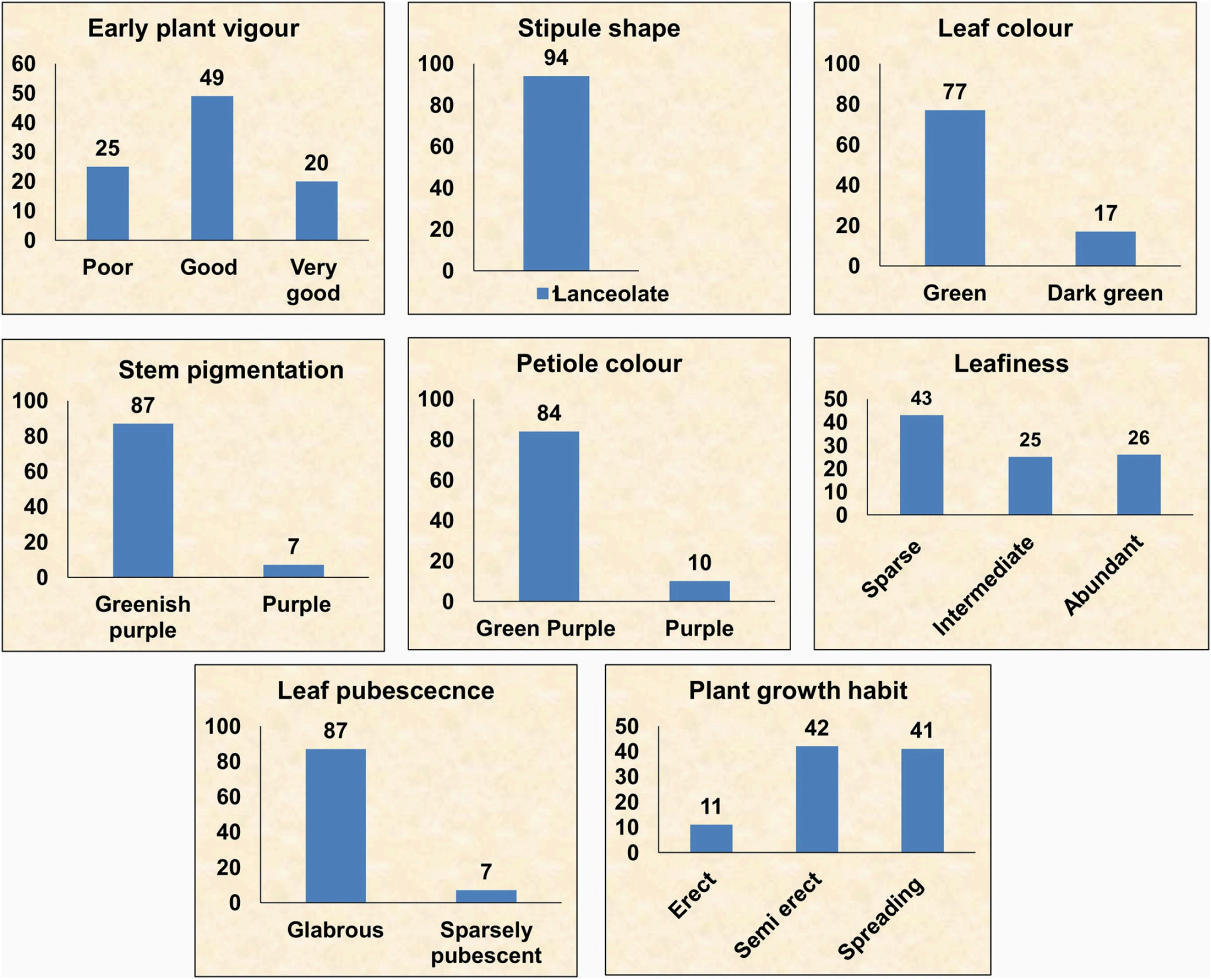
Variation in qualitative traits at vegetative stage among 94 accessions of *V*. *stipulacea*.

The traits studied at the vegetative stage were early plant vigour, stipule shape, leaf colour, branch pigmentation, petiole colour, leafiness, leaf pubescence and plant growth habit. Fifty per cent of the accessions had good plant vigour 30 days after sowing (DAS). Stipule shape of all accessions was lanceolate. Green and dark green coloured leaves were observed. Further, the majority of the accessions (77 accessions) had green coloured leaves. Greenish purple pigmentation on the stem was recorded while greenish purple coloured petiole was observed on 84 accessions. Significant variation was observed for the leafiness traits among different accessions. A higher proportion (43 accessions) was of sparse leafy types, while the intermediate (25) and abundant (26) leafy type accessions were almost in equal numbers. Accessions with high biomass and foliage had green fodder potential. There was predominance of accessions with glabrous type of leaves, stem and petiole. As far as plant type is concerned, most of the accessions under study were spreading (41) and semi-erect (42) while, 11 accessions were erect type (Fig 4).

#### Pod formation and maturity stage

**Fig 5:**
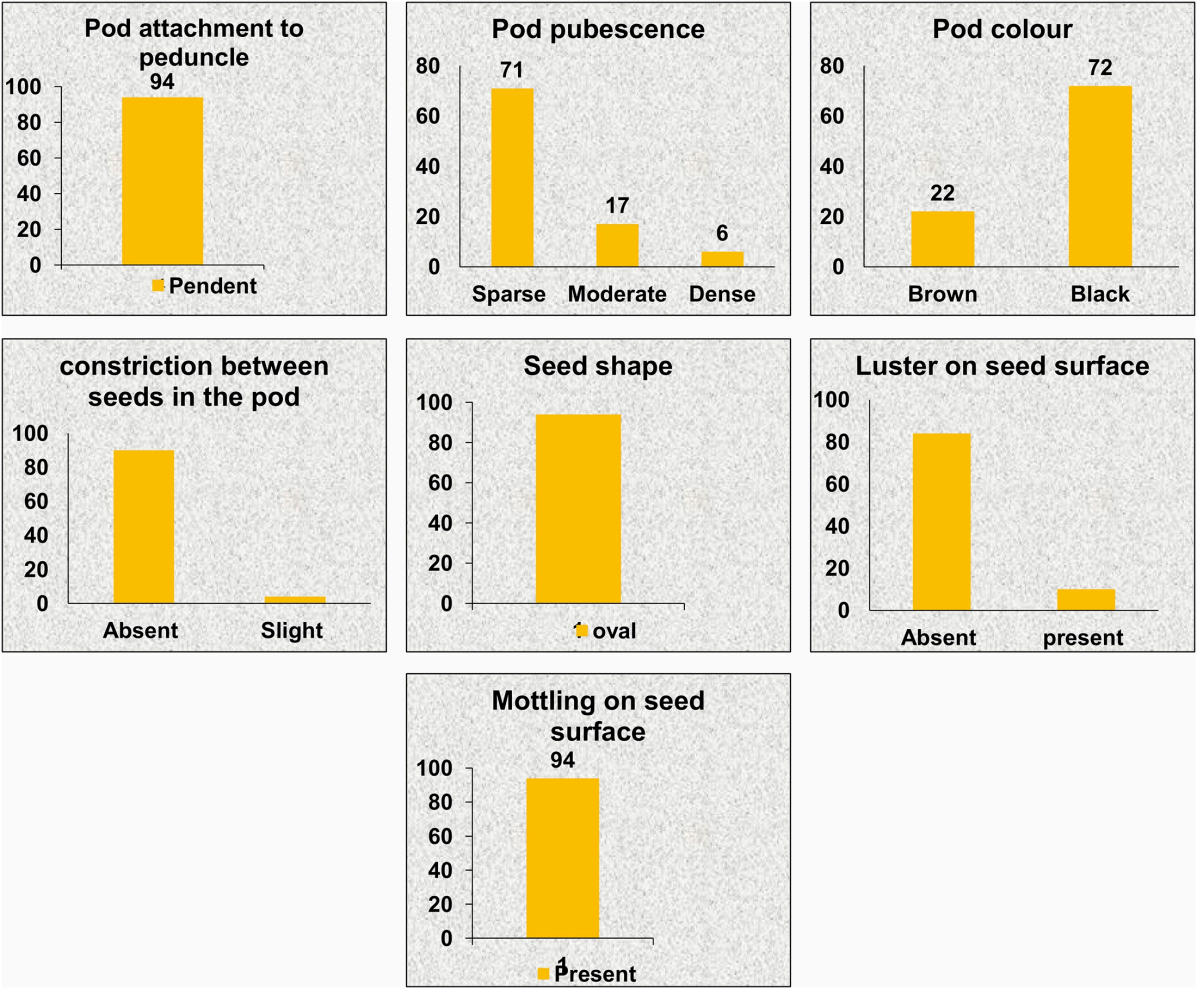
Variation in qualitative traits at pod formation and maturity stage among 94 accessions of *V*. *stipulacea*.

At pod formation and maturity stage, attachment of pod to peduncle, pod pubescence, constriction of pod between seeds, pod colour, seed colour, seed shape, lustre on seed surface and mottling on seed surface were recorded for all accessions. Pods of all accessions were pendently attached with the peduncle. There was a predominance of accessions with sparse hairs on pods whereas, for pod colour, black was more prevalent (72 accessions). All accessions had mottled, lustrous seed surface with oval seed shape (Fig 5).

*Germplasm characterization on the basis of morphological observations*. Based on two years’ evaluation, promising accessions were selected for the traits analyzed (Table 4). The accessions which started flowering early (less than 30 days) were IC251436, IC331436 IC331437. More than 50 cm. peduncle length was possessed by the accessions IC553518, IC550531, IC553557, IC553540, IC550532, IC553564. Accessions IC553526, IC553553, IC550538, IC553564, IC550533 had >6.5 cm terminal leaf length. Pod length was highest (5.40 cm) in IC622865, IC553560, IC550538, IC550553, IC553505, IC553523, and IC553509. Accessions with more than 13 numbers of seeds per pod were IC553529, IC622865, IC622867, IC550538 269 and IC553528.

**Table 4:** Characterization of germplasm accessions on the basis of morphological observations on desirable traits.

Trait	Promising accessions*
Peduncle length (more than 50 cm)	IC553518, **IC550531**, IC553557, IC553540, IC550532, IC553564
Green fodder potential	**IC550531**, IC550532, IC550533, IC550536, IC550545, IC550548, IC550551, IC550553, IC553502, IC553527, **IC553528, IC553529**, IC553530, IC553531, IC553532, IC331453, **IC331454**, IC331456, **IC331457**, IC331610
Days to initial flowering (less than 30 days)	**IC331436, IC251436, IC331437**
Days to initial maturity (less than 60 days)	**IC331436, IC251436, IC331437**, IC622861, IC276983, **IC622865, IC622867**, IC622860, **IC331454, IC331457**
Number of seeds per pod (more than 13)	**IC553529, IC622865, IC622867, IC553528**

*Accessions in bold represent superiority for two or more traits.

### SSR characterization

Genetic diversity analysis using 33 SSRs revealed significant variation present in the *V*. *stipulacea* germplasm (Table 5). The allelic size of SSRs varied from 80 to 290 bp among 94 *V*. *stipulacea* accessions. SSRs generated a total of 116 alleles, ranging from 2 to 7 alleles with an average of 3.52 alleles per marker. The Maf ranged from 0.32 to 0.88 averaging 0.68 per locus wherein, highest Maf was reported in the marker, MgSSR172 while the lowest in MgSSR148. The GD among 33 SSRs varied from 0.22 to 0.78 averaging 0.45 per marker. The highest GD was reported in MgSSR148, while the lowest in MgSSR172. The observed heterozygosity varied from 0.00 to 0.27 with a mean of 0.03 per marker. The PIC ranged from 0.20 (VR147) to 0.74 (MgSSR148) with mean of 0.40 per marker. Nine SSRs viz., VR024, VR025, VR393, VM27, MgSSR56, MgSSR148, MB122A, AB128100 and PvM13b had PIC value of >0.5 for the *V*. *stipulacea* germplasm, which reveled their high discrimination power.

**Table 5:** Genetic variability indices of the SSRs among the set of *V*. *stipulacea* accessions.

SN	Primer ID	Sequence F-forward, R-Reverse	Allele Size	Maf	An	GD	Ho	PIC
1.	VR022	F-TCTCTTCTCTCTTCTCTCTTCTTCTTC R-TTGTGTCTGAGGCTATGTTGGT	210-290	0.79	4.00	0.36	0.01	0.34
2.	VR024	F-GCTCTAAAACACGAAAGGGGT R-TCATGGTGGAAGAAAAGCAA	120-140	0.47	3.00	0.60	0.00	0.51
3.	VR025	F-GCTGTGGTGTATTTACCTTGGG R-ATCCTCCGGTCATTATCTTGTG	80-120	0.53	4.00	0.64	0.04	0.59
4.	VR029	F-GTGGCTCACAAGGTAGTGCTAA R-GAGAGAAACAACCAACCAAAGG	200-230	0.76	3.00	0.39	0.15	0.36
5.	VR032	F-ATATCAGCCATTGTTGCTTTCC R- TTCCCAGTTCAGACAACCAAGT	230-290	0.69	4.00	0.47	0.00	0.42
6.	VR040	F-TGACAACATGGGAAGAAGAAGA R-ACACCAACACAAAAGCAAACAC	150-190	0.80	4.00	0.34	0.15	0.32
7.	VR095	F-CATGTGAGCTACCTTTCAACA R-CAAGGACTGCTATATCCAAGGC	120-160	0.74	3.00	0.41	0.00	0.38
8.	VR102	F-GCTCCAACACTCACTCACAAAC R-CAGAAATGCAGGAAAAGAGAGG	120-140	0.86	2.00	0.24	0.00	0.21
9.	VR108	F-TGCATCTTTATTGAGTTCCGTG R-GTTTTGGGGTGAATGTTGGATA	220-250	0.59	3.00	0.55	0.00	0.48
10.	VR111	F-GGTGTTGTTGTTGAGGAATGAA R-AACATTGAGGACCCACATATCC	160-200	0.76	4.00	0.40	0.00	0.38
11.	VR147	F-CCATGTGTGTGAATGTGAGTGA R-CCTTTGATTTTGTGGGATGTGT	90-120	0.87	4.00	0.23	0.01	0.20
12.	VR256	F-GCTGTGGTGTATTTACCTTGGG R-ATCCTCCGGTCATTATCTTGTG	100-120	0.71	3.00	0.43	0.00	0.38
13.	VR393	F-TGGCACTTTCCATAACGAATAC R-ATCAGCCAAAAGCTCAGAAAAC	130-160	0.50	3.00	0.62	0.00	0.55
14.	VR400	F-ATCATAGATAGGGGACCAACCC R-ATCTTAGGGAGTCTTCGAGGGA	130-160	0.81	3.00	0.33	0.00	0.30
15.	VR413	F-GAGAAACCTTGGAGTTGGAGG R-GCCTGTCAAGAAGGAACCTAAA	110-130	0.55	2.00	0.49	0.00	0.37
16.	VM24	F-TCAACAACACCTAGGAGCCAA R-ATCGTGACCTAGTGCCCACC	150-170	0.66	2.00	0.45	0.00	0.35
17.	VM27	F-GTCCAAAGCAAATGAGTCAA R-TGAATGACAATGAGGGTGC	190-280	0.38	6.00	0.74	0.13	0.70
18.	MgSSR25	F-CCATCATTCTTGCAATTGCG R-AGCAACGAGACCTTGTTGCC	180-210	0.80	4.00	0.34	0.01	0.31
19.	MgSSR56	F-CTAAATGCAACAACACATGACACC R-ATTTGTATGGGTGCGACACC	150-190	0.49	4.00	0.62	0.01	0.54
20.	MgSSR63	F-TCAGGATATGCTCACCGTGC R-CCACCTCCTAGGGAGTGTCC	180-210	0.78	3.00	0.37	0.00	0.34
21.	MgSSR142	F- TTTTGCATTGTTTTGCAGGG R-TAGCCTCTAATCGCTCTGGC	100-190	0.63	4.00	0.53	0.00	0.47
22.	MgSSR148	F-AGCTACACAGATCACCTGGTGC R-TCGGAGTGGAGAAGAGAGTCG	150-280	0.32	7.00	0.78	0.27	0.74
23.	MgSSR172	F-CGTGCGATCACACATGTGC R-CCTATTTTATTAGTTGCACCACC	100-130	0.88	4.00	0.22	0.12	0.21
24.	MB122A	F-TGGTTGGTTGGTTCACAAGA R-CACGGGTTCTGTCTCCAATA	190-220	0.54	4.00	0.61	0.02	0.55
25.	MB323B	F-GCTATGCTATCGACACTGACTGA R-GCGCAAAGAGAGAGAGAGAGA	220-250	0.80	3.00	0.34	0.00	0.31
26.	AB128093	F-CCCGATGAACGCTAATGCTG R-CGCCAAAGAAACGCAGAAC	180-200	0.79	3.00	0.36	0.00	0.33
27.	AB128100	F-CATCTTCCTCACCTGCATTC R-TTTGGTGAAGATGACAGCCC	140-190	0.41	5.00	0.72	0.02	0.67
28.	PvM03	F-CCGCCTTCTTCTTCTTCTTC R-CGGCGAGTCATCTTTTCC	180-230	0.78	4.00	0.37	0.01	0.34
29.	PvM13b	F-GAGAAGCCGCAGAGAGGA R-AGATGCCGCGAACAGAAC	210-270	0.47	4.00	0.67	0.02	0.62
30.	BMD-18	F-AAAGTTGGACGCACTGTGATT R-TCGTGAGGTAGGAGTTTGGTG	110-130	0.82	2.00	0.30	0.00	0.25
31.	BMD-26	F-CTTGCCTTGTGCTTCCTTCT R-TCCATTCCCAACCAAGTTTC	110-120	0.74	2.00	0.39	0.14	0.31
32.	BMD-48	F-CCCCACCAACTCTTTCTTCC R-CAGAATTGACTTGGCGAGAA	120-130	0.74	2.00	0.38	0.00	0.31
33.	BMD-47	F-ACCTGGTCCCTCAAACCAAT R-CAATGGAGCACCAAAGATCA	130-190	0.86	4.00	0.25	0.00	0.24
**Mean**				**0.68**	**3.52**	**0.45**	**0.03**	**0.40**

**Where**; Maf-major allele frequency, An-Allele number, GD-gene diversity, Ho-observed heterozygosity, PIC- polymorphic information content.

### Phylogenetic studies

**Fig 6:**
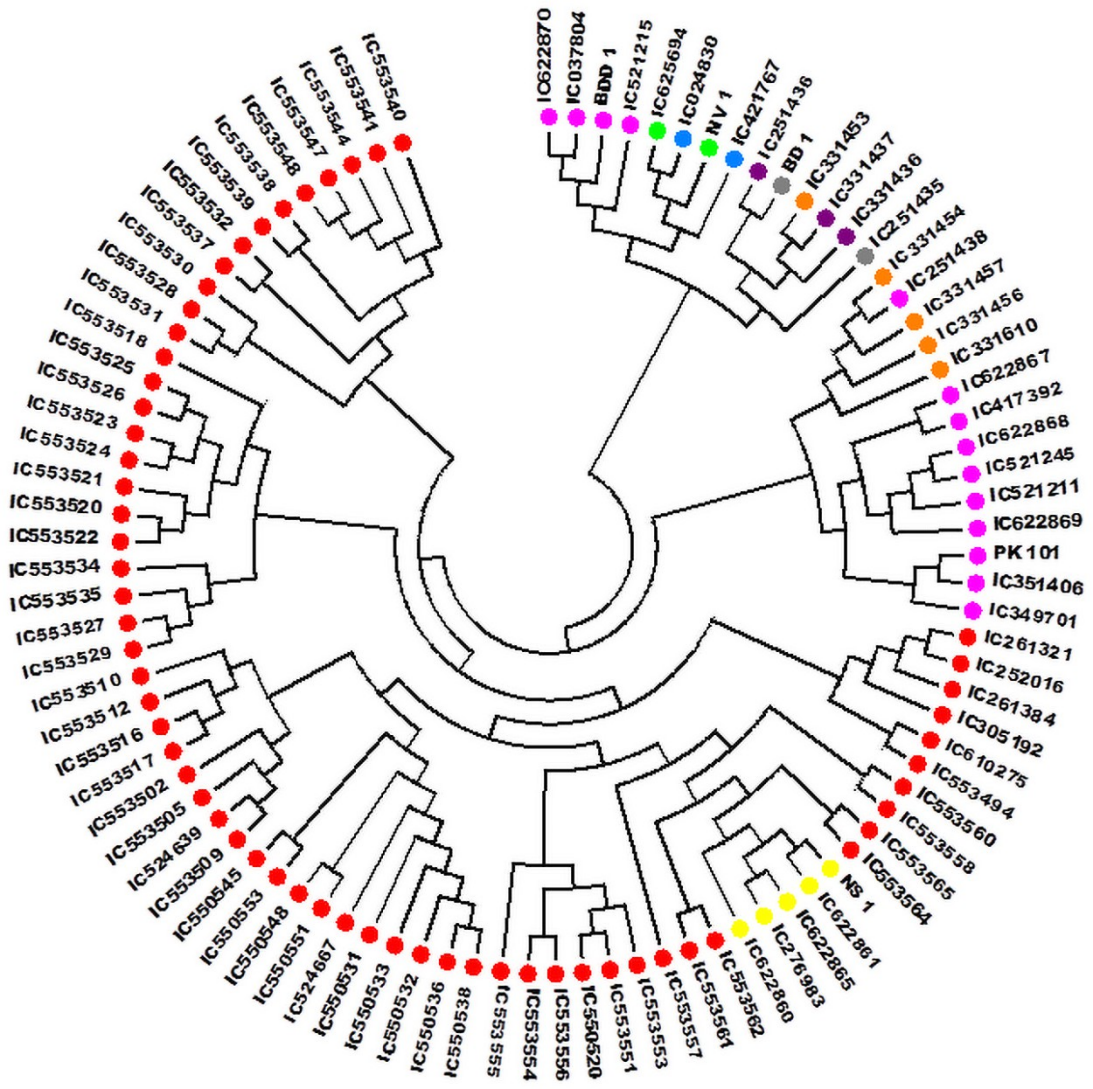
Dendrogram based on nei genetic diversity using Neighbor joining tree method of clustering, depicting genetic diversity and relationship between 94 accessions of *V*. *stipulacea* as revealed by polymorphism of 33 SSRs.

The Neighbor-joining (N-J) tree genetically differentiated 94 accessions of *V*. *stipulacea* into two major clusters (Fig 6). The N-J tree was further simplified into seven clades for its better explanation and understanding. Clade 1, 2, 3 and 5 contained accessions belonging to Andhra Pradesh while Clade 4 possessed accessions of Andhra Pradesh along with the accessions of Madhya Pradesh. Ten accessions of Tamil Nadu and 4 of Chhattisgarh were grouped in the Clade 6. Clade 7 had the mixture of accessions from different geographical regions *viz*., Tamil Nadu, Gujarat, Kerala, Karnataka, Odisha and Chhattisgarh.

### Population structure analysis

**Fig 7:**
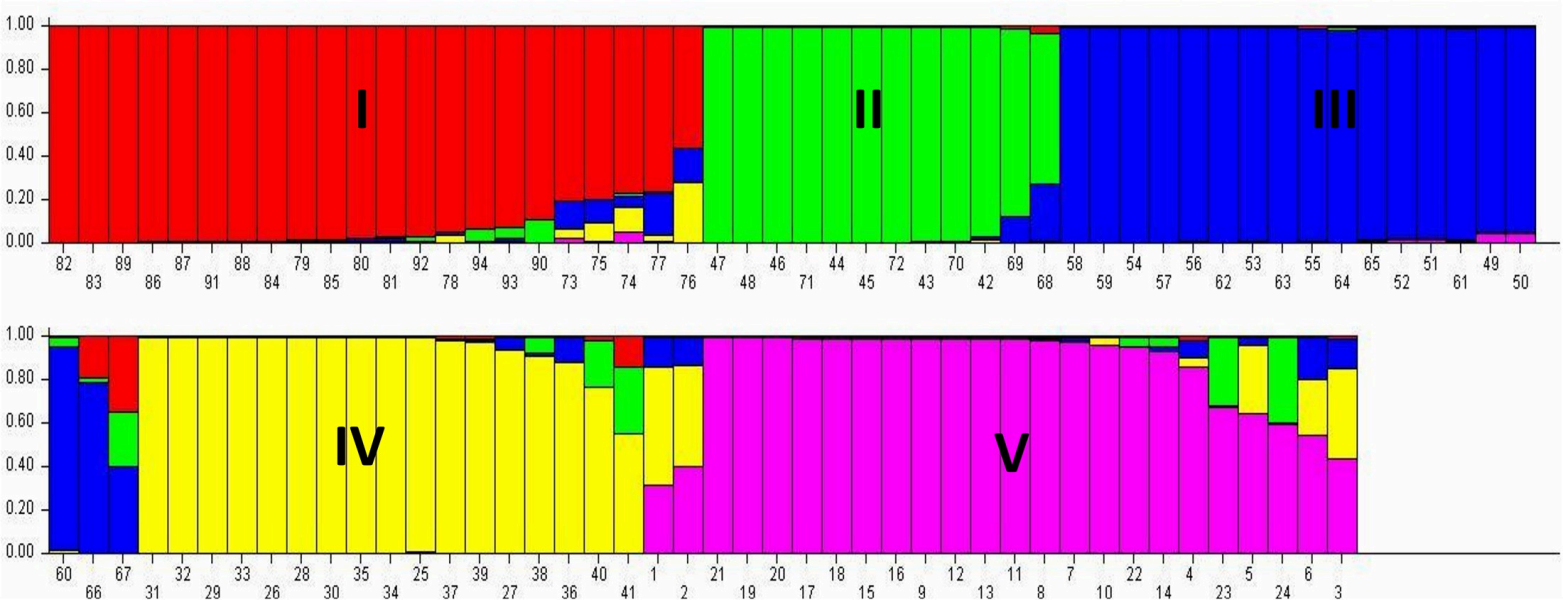
Barplot of population structure (K = 5) of 94 accessions of *V*. *stipulacea* based on 33 SSRs. Each individual bar represents an accession. The serial number of the *V*. *stipulacea* accessions in the barplot follows Table 1.

The 94 accessions of *V*. *stipulacea* were grouped into five population genetic groups recording the most probable number of K (S3 Fig) and grouping of each individual was illustrated using bar plot diagram. Of the 94 accessions, 80 (85.10%) shared their ancestry with one of the five population groups, while the remaining 14 (14.89%) were designated as admixed forms with varying levels of memberships shared among the five genetic populations. The population I contained 22 accessions with 19 pure and 3 admixtures. The population II comprised of 12 accessions with 11 pure and 1 admixture forms. The population III comprised of 17 pure and 2 admixture accessions. Population IV contained 15 pure and 2 admixture accessions. Subsequently, the population V comprised a total of 24 accessions with 17 pure and 7 admixtures (Fig 7).

The Fsts values of the *V*. *stipulacea* populations were computed, the mean Fst values of the populations being 0.3362, 0.4473, 0.6385, 0.6902 and 0.5324 for populations I to V, respectively, while the mean alpha value was 0.0340 (S4 Table).

The allele-frequency divergence of population I with population II, III, IV and V was 0.2093, 0.2184, 0.2778 and 0.2182, respectively. The allele-frequency divergence of population II with population III, IV and V was 0.3366, 0.3447 and 0.3399, respectively. Subsequently population III had 0.1651 and 0.1056 allele-frequency divergence with population IV and V, respectively. Population IV and V had 0.1976 allele-frequency divergence (Table 6).

**Table 6:** Allele-frequency divergence among populations of *V*. *stipulacea* accessions.

	**Pop1**	**Pop2**	**Pop3**	**Pop4**	**Pop5**
**Pop1**	-	0.2093	0.2184	0.2778	0.2182
**Pop2**	0.2093	-	0.3366	0.3447	0.3399
**Pop3**	0.2184	0.3366	-	0.1651	0.1056
**Pop4**	0.2778	0.3447	0.1651	-	0.1976
**Pop5**	0.2182	0.3399	0.1056	0.1976	-

### AMOVA and PCoA

AMOVA of five *V*. *stiupulacea* populations (as per the model-based population structure analysis) described 39% variations among the populations; 55% variations among individuals and 06% within individuals (S5 Table).

**Fig 8:**
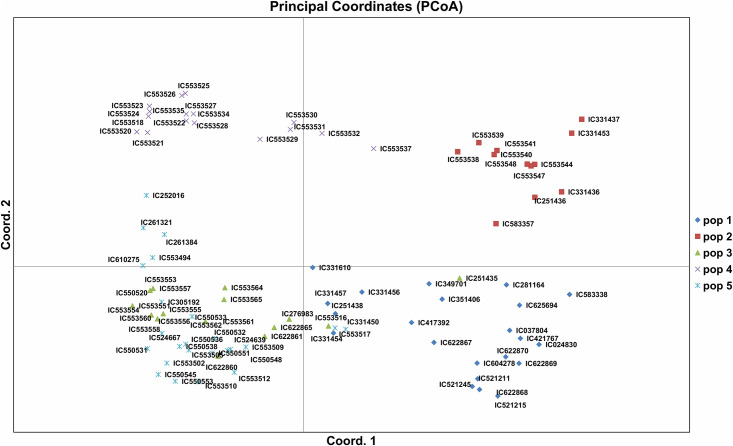
Principal Coordinate Analysis (PCoA) of 94 accessions of *V*. *stipulacea* based on 33 SSRs.

Besides, principal coordinate analysis (PCoA) showed high genetic variation among 94 accessions of *V*. *stipulacea*. Cumulative variation of three axes of PCoA was 40.01%, wherein first axes explained 20.04%; second 10.58% and third 9.39% of genetic variation. In the PCoA, the different population groups were denoted by five different colours, which were distributed over the coordinates (Fig 8).

## Discussion

Wild and underutilized germplasm offers novel sources of diversity hitherto not found in the domesticated species and consequently offers further opportunities of selection for agronomic traits [39]. Assessment of genetic diversity generates knowledge which is valuable for managing and conserving genetic resources [40]. In addition, it helps in identifying contrasting parental materials which can be utilized in breeding programmes. This genetic diversity can also be deployed to develop inbred lines with required genes and/or gene complexes introgressed from diverse and underutilized germplasm. The information of genetic diversity of germplasm has been extensively recognized in the selection of genotypes for yield and yield attributing characters, resistance to biotic and abiotic stresses as well as for nutritional improvement of crop species [41]. In the same line of thought, we carried out genetic diversity analysis of potential legume crop *V*. *stipulacea* based on morphological traits and microsatellite markers intending to identify and select diverse genotypes for use in further breeding programs.

Phenotypic evaluation of the genetic resources based upon morphological descriptors remains the first step for the evaluation, description and categorization of germplasm collections to augment their utilization in crop breeding program [23, 42]. The morphological characterization of wild *Vigna* species has been carried out by several earlier workers [Reviewed by Gore et al., (2019) 11]. All such studies suggested that wide range of exploitable variation is available in wild *Vigna* species. *V*. *stipulacea* was proposed as a candidate for neodomestication for disease and pest resistance by Tomooka et al. (2014) [5], whereas resistance to diseases and pests this species was also reported in several other reports (Reviewed by Pratap et al., 2021) [43]. Nutritional potential of *V*. *stipulacea* was unveilded by Gore et al., (2021b) [44]. Pandiyan et al., (2010) [45] reported that *V*. *stipulacea* is very closely related and crossable to *V*.*radiata*.

In the present study, stipule length varied from 1.10 to 2.63 mm with a mean of 1.70 mm. Among different morphological traits, the stipule length has been reported as one of the key characters for identification of *V*. *stipulacea* [46, 47]. Long peduncles are advantageous in getting the pods above the canopy. This helps in reducing the damage of pods by the pod borer and other insects and is also helpful at the time of harvesting [48]. This trait may be transferred to cultivated *Vigna* for facilitating mechanical harvesting, especially in mungbean,which is highly desirable keeping in view the increasing cost of labour and drudgery involved in manual picking of mature pods. In our study, a high coefficient of variation (30.22%) was observed for peduncle length. The longest peduncle length (63.0 cm) was recorded in the accession IC553564. Accessions with longer peduncles can be utilized for *Vigna* improvement programme and also for fibre production. Long peduncle of cowpea cultivar (*V*. *unguiculata* cv. *textilis*) was reported to be used for fibre in Africa [49]. Among the quantitative traits, days to initial flowering and maturity were the most important traits of observation. Crop duration has a direct impact of fitting a cultivar in a cropping system, and early duration varieties of mungbean have been developed which can fit well in short-season windows, thereby offering the opportunities of horizontal increase in area [39]. Days to initial flowering significantly varied among accessions with the mean value of 37 days. Likewise, days to initial maturity also recorded significant variation and ranged between 49 to 76 days. Among all accessions, IC331436 matured at the earliest as it matured within 49 days after sowing. Other accessions namely, IC251436, IC331437, IC622861, IC276983, IC622865, IC622867, IC622860, IC331454, IC331457 matured in <60 days. Therefore, these accessions may be utilized as donors for developing early maturing genotypes in green gram and black gram. Shorter duration cultivars can help in avoiding the terminal heat stress, especially in spring crop and also the terminal rains at the time of harvest during the rainy season in India [50]. Therefore, accessions with early maturity can be tremendously useful in expanding area in the northern and central part of India during the summer season. The wide variability for pod length was also observed, pod of *V*. *stipulacea* is harder and more resistant to the attack by stink bug as compared to the green gram and black gram [14]. Accessions IC553529, IC622865 and IC622867 recorded 15 number of seeds per pod, followed by IC553528 (14 seeds) and therefore, these were observed as the most promising for hybridization programmes towards increasing number of seeds per pods and ultimately seed yield as well as for selecting for direct use as a crop in domestication. Several earlier studies have reported significant positive correlation between number of seeds/pod and grain yield in mungbean [51], blackgram [52] and cowpea [53]. In *V*. *sublobata*, *V*. *radiata* and *V*. *trilobata*, up to 13 number of seeds per pod were recorded [23]. In the cultivated *Vigna* species like mungbean and urdbean usually 9–11 seeds per pod are observed and increasing 2–3 seeds per pod may increase the seed yield by upto 10 per cent. Therefore, the accessions with more number of seed per pod can be utilized in improving the cultivated *Vigna* species. These results of correlation analysis are in consonance with those of Bisht et al., 2005 [23] on studies of different wild *Vigna* species. An intermediate type of germination was most common in *V*. *stipulacea* which is the stage between hypogeal and epigeal germination [54]. In most of the accessions cotyledons were above the ground surface but hypocotyl was underground. These observations were similar to reports of Dixit, 2014 [46], Dixit et al., 2011 [55]. Primary leaf attachment is petiolate type, Tomooka et al., 2002 [56] suggested that this trait can be useful for taxonomic delineation among closely related species.

Spreading and semi-erect type of plant growth habit was most prevalent among accessions. Earlier studies also reported spreading type plant growth habit of *V*. *stipulcaea* [47, 56]. Erect type of plant is important for mechanical harvesting as well for use as fodder crops. In our study, 11 accessions were found to be of erect plant habit that can be utilized in the *Vigna* improvement programme. There was a predominance of accessions with sparse hairs on pods. Pubescence plays a vital role in protecting the crop from different biotic and abiotic stresses along with its adaptation mechanism. Pod pubescence as unique character in *Vigna unguiculata* (L.) Walp was reported by Tripathi et al., 2021 [57]. Among the pods black colour was more prevalent at maturity (72 accessions). Pod colour acts as a morphological marker and may be deployed in quality seed production programmes to monitor the mixture of other varieties. All accessions had mottled, lustrous seed surface.

Assessment of genetic resources of *V*. *stipulacea* for identification of useful germplasm is essential for enhancing their utilization by the plant breeders towards genetic improvement of different *Vigna* crops and also for genebank managers to refine genetic resource management strategy. This study led to the identification of desirable germplasm lines for specific traits like long peduncle, green fodder potential, early maturity and more number of seeds per pod. More interestingly, accessions with multiple traits were also identified which included, for example, green fodder potential and more number of seeds per pod (IC553528 and IC553529); long peduncle length and green fodder potential (IC550531); early flowering and early maturity (IC331436, IC251436, IC331437) and early maturity and more number of seeds/pods (IC622865, IC622867). All the identified trait-specific germplasm was validated again in *Kharif* 2020 at three locations *viz*., IIPR, Kanpur, NBPGR, main campus, New Delhi, and NBPGR, Issapur Farm. Therefore, the promising accessions identified through this study can be used in *Vigna* breeding programs to select high yielding varieties with enhanced resistance to biotic stresses and having green fodder potential. Simultaneously, the potential of outstanding accessions can also be exploited by their direct introduction in the farmers’ fields for their multifarious uses. Nonetheless, keeping in view that the study involves an evaluation of an Indian germplasm, there is a further need of comprehensive evaluation of a large number of global germplasm lines to identify trait-specific potential germplasm adapted in India and elsewhere.

Among various molecular markers, microsatellite markers (SSRs) were used by many earlier workers and recognized as highly informative and efficient for studying genetic diversity due to their co-dominant nature, high reproducibility, high rate of cross- transferability, easiness in scoring [58]. In the past, Pratap et al., (2015) [1]; Kumari et al., (2021) [21]; Pratap et al., (2021) [43] have efficiently characterized the *Vigna* germplasm using a set of SSR loci and decoded the extent of genetic variability. In the present study, 33 polymorphic primers generated a total of 116 alleles with an average of 3.52 bands per primer. The mean value of gene diversity was 0.53. Gene diversity is a vital indicator of the effectiveness of a primer. Major allele frequency ranged from 0.27 to 0.90. PIC ranged from 0.18 to 0.82 averaging 0.49. The PIC value of primers indicated greater diversity within and between studied *Vigna* accessions and also revealed that selected primers are very informative and can be utilized in the further studies on *Vigna* genotypes. In a similar study, Pratap et al., (2015) [1] studied 53 *Vigna* accessions *viz*., 41 wild and 12 cultivated lines. The MAF varied from 0.16 to 0.65 with average PIC of 0.79. Similar observations were recorded [59] by studying 15 SSR markers on 65 mungbean genotypes for genetic diversity analysis. Seventy genotypes of the *Vigna* genus for RAPD, URP and SSRs were studied and reported that SSRs detected 211 polymorphic bands in the *Vigna* species [29]. More number of alleles per locus is one of the important criteria for selection of the marker to be utilized in the assessment of genetic variability [60]. Primers with higher PIC value and multiple-locus amplification have been recommended for germplasm characterization, evaluation, phylogenetic studies and breeding applications. In the current investigation, the PIC value of primers indicated greater diversity within and between studied germplasm and also revealed that selected primers are very informative and can be utilized in the further studies on *Vigna* genotypes.

The N-J tree clustered 94 accessions of *V*. *stipulacea* into seven clades which suggested a high degree of their genetic divergence. The SSRs amply discriminated the accessions under study. Strikingly, the overall clustering of the *V*. *stipulacea* accessions was not as per their geographical origins, as many of the accessions from the same geographical origin (for example accession from Tamil Nadu) did not group together. Earlier, Pratap et al. (2015) [1] also reported that accessions of *V*. *trilobata* (a close relative of *V*. *stipulacea*) grouped in different clusters despite of same geographic origin and ascribed it to their collection across the agro-ecological regions and also that some of them might have experienced natural hybridization in due course of time which explains their admixture status. Studies on 692 accessions of mungbean by Gwag et al., (2010) [61] collected from 27 countries reported that due to germplasm exchange between countries, genetic associations in accession under study were not associated with geographical origin. Genetic diversity not always follows the geographical origin of germplasm which may be due to extensive germplasm exchange from different geographical regions [62]. The 3-D PCA grouping patterns were also in general agreement with the grouping described in the dendrogram. Similarly, Pratap et al., (2015); Dikshit et al., (2007) [1, 60] while studying the *Vigna* species also reported that the results of the cluster analysis were comparable to the PCA. Population structure analysis based on the model is one of the excellent tools for the assessment of the genetic diversity in any plant species [63], which could be magnificently applied in conservation and utilization of the collected germplasm [64]. In the recent past, population structure analysis was extensively utilized for genetic diversity analysis of the *Vigna* germplasm. For example, 53 accessions of *Vigna* species were genetically grouped into five groups using the model-based population structure analysis [1]. Similarly, AMOVA also described high genetic variability among the populations, among and within the individual of the populations. Besides, PCoA partly revalidated this genetic grouping of the *V*. *stipulacea* accessions. The potential for utilization of different wild *Vigna* species has been described by Takahashi et al., (2016) [65] based on estimation of phylogenetic positions by using rDNA-ITS (nuclear) and *atpB-rbcL* (chloroplast) spacer regions.

Our result revealed significant variations in the morphological traits and microsatellite DNA polymorphisms in *V*. *stipulacea* accessions. The high allelic diversity in the analyzed germplasm lines indicated that the genetic spectrum was relatively wide. *V*. *stipulacea* has a great potential which can be exploited for improving other domesticated *Vigna* species by broadening their genetic base which can be useful to fight with biotic and abiotic stresses, especially in the climate change scenario. The study identified superior accessions for several traits which can be deployed in hybridization programme for improvement of mungbean and black gram.

## Supporting information

S1 Table Details of qualitative traits, states, code and stage of recording observation. (DOCX)

S2 Table Primer used for initial screening to identify polymorphic markers on a panel of 20 representative accessions. (DOCX)

S3 Table Mean value of all the quantitative traits. (DOCX)

S4 Table Mean value inferred from model-based approach. (DOCX)

S5 Table Analysis of molecular variance of 33 SSRs among 94 *V*. *stipulacea* accessions. (DOCX)

S1 Fig Field view of *V*. *stipulacea* characterization experiment at IIPR, Kanpur, India. (TIF)

S2 Fig Variability in morphological traits *viz*. a) peduncle length; b) leaf shape and size; c) pod length.
(TIF)

S3 Fig Estimation of population using LnP (D) derived delta k for k from 2 to 10. (TIF)
