# Supplementary material for: Insights into the genetic diversity of an underutilized Indian legume, Vigna stipulacea (Lam.) Kuntz., using morphological traits and microsatellite markers
Source: PLoS One. 2022 Jan 19;17(1):e0262634. doi: 10.1371/journal.pone.0262634 (PMC8769370; doi:10.1371/journal.pone.0262634)
Supplement: S1 Table — (DOCX) [file pone.0262634.s001.docx]

**Table S1. Details of qualitative traits, states, code and stage of recording observation**

| **S. N.** | **Traits** | **State** | **Code** | **Observation** |
| --- | --- | --- | --- | --- |
|  | Seed Germination habit | Epigeal  Intermediate  Hypogeal | 1  2  3 | Recorded at germination stage |
|  | Hypocotyl colour | Green  Purple  Greenish purple  Other | 1  2  3  99 | Recorded after 10 days of emergence |
|  | Attachment of primary leaves | Sessile  Sub-sessile  Petiolate | 1  2  3 | Recorded at two leaf stage |
|  | Early plant vigour | Poor  Good  Very good | 1  2  3 | Measured after 20-25 days of sowing |
|  | Stipule shape | Ovate  Lanceolate  Others | 1  2  99 | Recorded at 50% flowering |
|  | Leaf colour | Light Green  Green  Dark Green | 1  2  3 | Recorded at 50% flowering |
|  | Branch pigmentation | Green  Greenish purple  Purple | 1  2  3 | Recorded at 50% flowering |
|  | Petiole colour | Green  Greenish purple  Purple  Dark purple | 1  2  3  4 | Recorded at 50% flowering |
|  | Leafiness | Sparse  Intermediate  Abundant | 1  2  3 | Recorded at 50% flowering |
|  | Leaf pubescence | Glabrous  Pubescent | 1  2 | Recorded at 50% flowering |
|  | Plant growth habit | Erect  Semi Erect  Spreading  Others | 1  2  3  99 | Recorded when the first pod changes colour |
|  | Attachment of pod to peduncle | Erect  Pendent  Others | 1  3  99 | Recorded at maturity stage |
|  | Pod pubescence | Glabrous  Sparsely pubescent  Moderately pubescent  Densely pubescent | 1  2  3  4 | Recorded when the first pod changes colour |
|  | Pod colour | Straw  Brown  Black  Other | 1  3  4  99 | Recorded at maturity stage |
|  | Constriction between seeds in the pod | Absent  Slight  Pronounced | 1  2  3 | Recorded when the first pod changes colour |
|  | Seed shape | Round  Oval  Drum  Others | 1  2  3  99 | Recorded after harvesting the seeds |
|  | Lustre on seed surface | Absent  Present | 1  2 | Recorded after harvesting the seeds |
|  | Mottling on seed surface | Absent  Slight  Intermediate  Heavy | 1  2  3  4 | Recorded after harvesting the seeds |
