# Supplementary material for: Insights into the genetic diversity of an underutilized Indian legume, Vigna stipulacea (Lam.) Kuntz., using morphological traits and microsatellite markers
Source: PLoS One. 2022 Jan 19;17(1):e0262634. doi: 10.1371/journal.pone.0262634 (PMC8769370; doi:10.1371/journal.pone.0262634)
Supplement: S2 Table — (DOCX) [file pone.0262634.s002.docx]

**Table S2. Primer used for initial screening to identify polymorphic markers on a panel of 20 representative accessions**

| S.N | Primer | Sequence |
| --- | --- | --- |
|  | mgSSR 25 | F-CCATCATTCTTGCAATTGCG  R-AGCAACGAGACCTTGTTGCC |
|  | mgSSR 56 | F-TAAATGCAACAACACATGACACC  R-ATTTGTATGGGTGCGACACC |
|  | mgSSR 63 | F-TCAGGATATGCTCACCGTGC  R-CCACCTCCTAGGGAGTGTCC |
|  | mgSSR 142 | F- TTTTGCATTGTTTTGCAGGG  R-TAGCCTCTAATCGCTCTGGC |
|  | mgSSR 148 | F-AGCTACACAGATCACCTGGTGC  R-TCGGAGTGGAGAAGAGAGTCG |
|  | mgSSR 157 | F-CCATGGTCATAATTTTGAATGGG  R-GCAATTCCGTGTTCGTTGGG |
|  | mgSSR 172 | F-CGTGCGATCACACATGTGC  R-CCTATTTTATTAGTTGCACCACC |
|  | mgSSR 170 | F-GCCTTATAAAAAATCGGACG  R-TACACGCGTGCACGAACAGG |
|  | mgSSR 173 | F-AGCATTGAGAGAGAACGTAGGG  R-CTCTTTTCTCTCTTTCTTCCTCC |
|  | mgSSR 177 | F-AAAGAGTTGAGTTGACAAAAGCG  R-AACACTCTAATTGCTTCTCC |
|  | mgSSR 229 | F-TTATGCAGTTCTTGAATGAGGG  R-GTAGTCTCTCTTCCTTCTCTCGC |
|  | mgSSR 240 | F-ATTTGCACAGTCAGGAAAAG  R-GCCTATCCTAAACATCACAAAC |
|  | mgSSR 244 | F-ACTGTTATTCCGACAACCTATC  R-CTTCGTTTTCTTCTTCCTCAC |
|  | VR-040 | F-TGACAACATGGGAAGAAGAAGA  R-ACACCAACACAAAAGCAAACAC |
|  | VR-102 | F-CATGTGAGCTACCTTTCAACA  R-CAAGGACTGCTATATCCAAGGC |
|  | VR-108 | F-GCTCCAACACTCACTCACAAAC  R-CAGAAATGCAGGAAAAGAGAGG |
|  | VR-111 | F-TGCATCTTTATTGAGTTCCGTG  R-GTTTTGGGGTGAATGTTGGATA |
|  | VR-140 | F-GGTGTTGTTGTTGAGGAATGAA  R-AACATTGAGGACCCACATATCC |
|  | VR-147 | F-CCATGTGTGTGAATGTGAGTGA  R-CCTTTGATTTTGTGGGATGTGT |
|  | VR-256 | F-GCTGTGGTGTATTTACCTTGGG  R-ATCCTCCGGTCATTATCTTGTG |
|  | VR-303 | F-AGACGAAGAAGAAAACGCAGAC  R-CCTCACACACAACACAACAGAA |
|  | VR-304 | F-GAAGCGAAGAAGCCATAGAAAA  R-CCTCACACACAACACAACAGAA |
|  | VR-338 | F-ACTGAAGAGAATGGGTTAGGGG  R-TCACATTTGTTGGGTTGAAGAG |
|  | VR-393 | F-TGGCACTTTCCATAACGAATAC  R-ATCAGCCAAAAGCTCAGAAAAC |
|  | VR-400 | F-ATCATAGATAGGGGACCAACCC  R-ATCTTAGGGAGTCTTCGAGGGA |
|  | VR-413 | F-GAGAAACCTTGGAGTTGGAGG  R-GCCTGTCAAGAAGGAACCTAAA |
|  | VR-468 | F-AGCTGCCCCTCTTTACTTAGATTT  R-CGTCATTGCATACTTGAATTGG |
|  | AB-128100 | F-CATCTTCCTCACCTGCATTC  R-TTTGGTGAAGATGACAGCCC |
|  | AB-128079 | F-AGCGAGTTTCGTTTCAAG  R-GCCCATATTTTTACGCCCAC |
|  | AB-128093 | F-CCCGATGAACGCTAATGCTG  R-CGCCAAAGAAACGCAGAAC |
|  | MB-120 B | F-AGCCCTTCGTGCTAGGAAAT  R-CCCTACCGGTTGGTTGGT |
|  | MB-122 A | F-TGGTTGGTTGGTTCACAAGA  R-CACGGGTTCTGTCTCCAATA |
|  | MB-322 B | F-TCAGTCAGTGTCGATAGCATAGC  R-GACACAGAGAGAGAGAGAGAG |
|  | MB-323 B | F-GCTATGCTATCGACACTGACTGA  R-GCGCAAAGAGAGAGAGAGAGA |
|  | MB-323 A | F-TGACGGAGAGAGAGAGAGAGAG  R-TGCTTCCTTTTGTCTGAGTTAGAA |
|  | MB-738 A | F-CGCAAAGAGAGAGAGAGAG  R-CCCCCATCTGAAAGAAAGAG |
|  | VM-24 | F-TCAACAACACCTAGGAGCCAA  R-ATCGTGACCTAGTGCCCACC |
|  | VM-27 | F-GTCCAAAGCAAATGAGTCAA  R-TGAATGACAATGAGGGTGC |
|  | VR095 | F- GAAATGGGAGTTCAAAGAGGAA  R-TGGAGAAGTCTGGAAGAGAACC |
|  | VR011 | F-TGCATCTTTATTGAGTTCCGTG  R-GTTTTGGGGTGAATGTTGGATA |
|  | VR013 | F-GAAGTGGCGGAAGATTGATAAG  R-TAGATGGAAGGTAGAGGAATGA |
|  | VR015 | F-AAGATCACACACAACCAACCC  R-AATTAGTTCCACAGGCCAGATT |
|  | VR016 | F-AGGAGAAATTGTTGTTGTTCGG  R-GTGTTGATTGTTAGGGAGGGAG |
|  | VR018 | F-ATACAAGGGCAGGTGTAGCAT  R-CAGAAAACTTCATCCCCAGCTA |
|  | VR021 | F-TTCCCTGTGTCCTTATATGTCC  R-GAGGATAGTGAATTTTGAAGGC |
|  | VR022 | FCTCTTCTCTCTTCTCTCTTCTTCTTC  R-TTGTGTCTGAGGCTATGTTGGT |
|  | VR024 | F-GCTCTAAAACACGAAAGGGGT  R-TCATGGTGGAAGAAAAGCAA |
|  | VR025 | F-GCTGTGGTGTATTTACCTTGGG  R-ATCCTCCGGTCATTATCTTGTG |
|  | VR029 | F-GTGGCTCACAAGGTAGTGCTAA  R-GAGAGAAACAACCAACCAAAGG |
|  | VR032 | F-ATATCAGCCATTGTTGCTTTCC  R-TTCCCAGTTCAGACAACCAAGT |
|  | J01263 | F-ATGCATGTTCCAACCACCTTCTC  R-GGAGTGGAACCCTTGCTCTCATC |
|  | PV-at001 | F-GGGAGGGTAGGGAAGCAGTG  R-GCGAACCACGTTCATGAATGA |
|  | PV-ag003 | F-ACGTACGAGTTGAATCTCAGGAT  R-GGTGTCGGAGAGGTTAAGGTTG |
|  | PV-ag005 | F-CTCACGTACGAGTTGAATCTCAG  R-ATCTGAGAGCAGCGACATGGTAG |
|  | SSR-IAC 127 | F-GAGGCTAGCCCAACTTA  R-AGCGCAAGACTTTACTACTC |
|  | SSR-IAC 188 | F-CCTGCCTTTGCCACTCCTC  R-CTCCTTCTACCCAGCCAAACC |
|  | SSR-IAC 195 | F-TGGACATCAAACAAACAAAAA  R-TGCATCGGCAGTTCATCA |
|  | DQ469392 | F-TCCCGATTTATAGTTCTCATTT  R-AGGGACCTCCTTCATCTC |
|  | DQ469393 | F-CATTGAGATTTGAGGTTTCGTT  R-AGGTATTTCCATCGTGCTTTTC |
|  | PvM03 | F-CCGCCTTCTTCTTCTTCTTC  R-CGGCGAGTCATCTTTTCC |
|  | PvM13b | F-GAGAAGCCGCAGAGAGGA  R-AGATGCCGCGAACAGAAC |
|  | PvM22 | F-ACTCTCACAATGGCGGAATC  R-GGCGTTTTCTCCCTCTTCTT |
|  | BMD-5 | F-CGTGGACTTGAATGGTTTCAG  R-TCCTTACCCTGTTCTGCTTCTC |
|  | BMD-6 | F-CATCGAATGCCCAAGAGAATA  R-CTCACTGTCTTCCATCCAAGC |
|  | BMD-8 | F-TTCATCCTCTCTCCCGAACTT  R-CTTTTGTGGCTGAGACATGGT |
|  | BMD-13 | F-TCATGCCTGAGAAAGGGTCT  R-CCCTGCATCAGAAGTCCAAT |
|  | BMD-18 | F-AAAGTTGGACGCACTGTGATT  R-TCGTGAGGTAGGAGTTTGGTG |
|  | BMD-23 | F-GGCTTGGTCCTCTCATTGAA  R-TGGAAATTACCACCATGCAA |
|  | BMD-26 | F-CTTGCCTTGTGCTTCCTTCT  R-TCCATTCCCAACCAAGTTTC |
|  | BMD-29 | F-CTTCACCGATCTGACAGCAG  R-TTTCTCCACTGGAACACTCG |
|  | BMD-31 | F-TGAAGAGGATCGCAAGGTTC  R-AGCCGAAACACTGTCCTTGT |
|  | BMD-48 | F-CCCCACCAACTCTTTCTTCC  R-CAGAATTGACTTGGCGAGAA |
|  | BMD-35 | F-TCTCTTCCTTACCCTGTTCTGC  R-GCGTGGACTTGAATGGTTTC |
|  | BMD-47 | F-ACCTGGTCCCTCAAACCAAT  R-CAATGGAGCACCAAAGATCA |
|  | BMD-12 | F-CATCAACAAGGACAGCCTCA  R-GCAGCTGGCGGGTAAAACAG |
|  | CEDG178 | F-CGGAAGAAGAACGCAGAGTG  R-GCATCAACAAGGACTTCTGC |
|  | CEDG254 | F-CGATGTCTCTTGCTTCAAGG  R-GTGAAGGACTAGCCAAGTTTG |
|  | CEDG263 | F-GATTGGGAATCTGCTGTTG  R-GTGATCCACACACAGTAC |
|  | CEDG050 | F-GGCAGAATCGTACAAGTG  R-GTCAGATTCTCGCTTGCATG |
|  | CEDG261 | F-GGTCCCAAAATCACCCAG  R-GGTTCATTTGGAGCACTGAG |
|  | CEDG010 | F-TGGGCTACCAACTTTTCCTC  R-TGAGCGACATCTTCAACACG |
|  | CEDG084 | F-CAACATTTCAACCTTGGGACAG  R-ATCAACTGAGGAGCATCATCGA |
|  | CEDG154 | F-GTCCTTGTTTTCCTCTCCATGG  R-CATCAGCTGTTCAACACCCTGTG |
|  | VR0135 | F-GCCCAGATTTGTTCATCCTAGA  R-ACTGTTTTGAGTGGGGAAAAGA |
|  | VR0155 | F-AAGATCACACACAACCAACCC  R-AATTAGTTCCACAGGCCAGATT |
|  | VR0163 | F-AGGAGAAATTGTTGTTGTTCGG  R-GTGTTGATTGTTAGGGAGGGAG |
|  | VR0188 | F-ATACAAGGGCAGGTGTAGCATC  R-CAGAAAACTTCATCCCCAGCTA |
|  | VR0216 | F-TTCCCTGTGTCCTTATATGTCC  R-GAGGATAGTGAATTTTGAAGGC |
|  | VR0222 | F-TCTTCTCTCTTCTCTCTTCTTCTTC  R-TTGTGTCTGAGGCTATGTTGGT |
|  | VR0223 | F-GCGTGATCGAGGCAGACTAT  R-GTGGGTAGCTCGGTAATAGCAC |
|  | VR0225 | F-CAGCAACAGAACTACAATCCCA  R-CGGCAATCCTCCTATATTCATT |
|  | VR0244 | F-GCTCTAAAACACGAAAGGGGT  R-TCATGGTGGAAGAAAAGCAA |
|  | VR0326 | F-GATGGCTCTGCATTGAAACC  R-GATCTTCCCAACTTTCCCTCTC |
|  | VR0357 | F-GCCCGATGTCCTAGCTTTTAG  R-CCTCAAAACAATCAGAACTCTCG |
|  | VR0375 | F-TCTCAGCATCTGTGGTGGTAGT  R-AGAATCCAACAACTCCTGCTTC |
|  | VR0398 | F-TGCTAAAGGTTTCCTCTCAACT  R-GAATGAAGTCACGCACACAA |
|  | VR0453 | F-TCTTTTCTATGTATGGCGCAAC  R-TTGGCTTTCGTATTTCCTCAGT |
|  | VR0487 | F-GGCAGGGAAGGAGGAAAA  R-CAGCCACAACAAGGCACA |
|  | AF350505 | F-CAGACATGCAAATTGGAAC  R-GGAGCACCAAAGATCATAGA |
|  | DQ345305 | F-GAGGCCAATCCCATAACTTT  R-AGCACCACATCAGAGATTCC |
