# Supplementary material for: Insights into the genetic diversity of an underutilized Indian legume, Vigna stipulacea (Lam.) Kuntz., using morphological traits and microsatellite markers
Source: PLoS One. 2022 Jan 19;17(1):e0262634. doi: 10.1371/journal.pone.0262634 (PMC8769370; doi:10.1371/journal.pone.0262634)
Supplement: S3 Table — (DOCX) [file pone.0262634.s003.docx]

**S3 Table. Mean value of all the quantitative traits**

| **Sr No** | **Acc. No** | **Stipule length** | **stipule width** | **Petiole length** | **Peduncle length** | **Branch length** | **Terminal leaf length** | **Pod length** | **Seed per pod** | **Days**  **to flow**  **ering** | **Days to first poding** | **Days**  **to maturity** | **Plant height** | **Chrolo**  **phyll content** | **Seed length** | **Seed width** |
| --- | --- | --- | --- | --- | --- | --- | --- | --- | --- | --- | --- | --- | --- | --- | --- | --- |
|  | IC252016 | 1.23 | 1.10 | 5.33 | 30.33 | 77 | 4.67 | 4.33 | 11.00 | 32 | 36 | 67 | 26.82 | 38.77 | 2.22 | 1.97 |
|  | IC261321 | 1.57 | 1.17 | 11.33 | 37.67 | 100 | 4.73 | 4.80 | 12.33 | 33 | 37 | 67 | 20.00 | 40.93 | 2.61 | 2.22 |
|  | IC261384 | 1.43 | 1.20 | 7.00 | 28.67 | 78 | 4.43 | 3.83 | 9.67 | 36 | 41 | 69 | 12.40 | 43.23 | 2.41 | 1.85 |
|  | IC305192 | 1.40 | 1.17 | 7.00 | 35.12 | 105 | 4.23 | 4.60 | 12.67 | 34 | 38 | 66 | 13.20 | 44.87 | 2.69 | 2.19 |
|  | IC553494 | 1.37 | 1.17 | 9.00 | 28.33 | 81 | 5.00 | 4.10 | 11.00 | 36 | 40 | 65 | 12.80 | 43.23 | 2.38 | 2.24 |
|  | IC610275 | 1.50 | 1.23 | 12.83 | 36.33 | 110 | 6.20 | 4.37 | 7.67 | 38 | 42 | 65 | 18.40 | 36.77 | 2.62 | 2.32 |
|  | IC524667 | 1.50 | 1.26 | 12.40 | 29.33 | 50 | 5.00 | 4.83 | 11.33 | 34 | 38 | 67 | 12.52 | 42.23 | 2.55 | 2.15 |
|  | IC550531 | 2.13 | 1.93 | 12.50 | 53.00 | 110 | 6.13 | 5.20 | 12.33 | 38 | 41 | 72 | 27.00 | 38.47 | 2.60 | 2.25 |
|  | IC550532 | 2.42 | 2.12 | 9.77 | 58.67 | 152 | 5.80 | 3.87 | 10.67 | 38 | 42 | 71 | 28.00 | 39.47 | 2.42 | 2.29 |
|  | IC550533 | 2.10 | 1.96 | 13.00 | 46.33 | 105 | 7.03 | 4.23 | 12.00 | 38 | 42 | 74 | 27.80 | 39.60 | 2.43 | 2.22 |
|  | IC550536 | 2.17 | 1.99 | 14.33 | 44.15 | 108 | 5.12 | 5.07 | 13.00 | 38 | 41 | 73 | 24.40 | 38.73 | 2.71 | 2.14 |
|  | IC550538 | 2.40 | 2.01 | 11.33 | 43.67 | 110 | 6.77 | 5.43 | 14.00 | 37 | 40 | 74 | 27.60 | 40.93 | 2.46 | 2.18 |
|  | IC550545 | 1.67 | 1.32 | 13.97 | 31.33 | 105 | 6.33 | 5.00 | 11.33 | 36 | 40 | 69 | 11.60 | 43.27 | 2.33 | 2.18 |
|  | IC550548 | 1.13 | 1.01 | 13.33 | 28.67 | 95 | 3.50 | 3.93 | 11.33 | 37 | 41 | 68 | 12.40 | 43.23 | 2.04 | 1.78 |
|  | IC550551 | 1.50 | 1.21 | 10.00 | 31.00 | 87 | 3.57 | 5.17 | 13.00 | 32 | 36 | 65 | 13.40 | 41.90 | 2.22 | 1.96 |
|  | IC550553 | 1.63 | 1.29 | 8.37 | 28.00 | 84 | 4.97 | 5.43 | 13.00 | 33 | 37 | 64 | 13.60 | 45.50 | 2.67 | 2.10 |
|  | IC553502 | 1.17 | 1.01 | 9.00 | 23.67 | 59 | 5.00 | 5.10 | 12.67 | 37 | 41 | 66 | 14.60 | 45.93 | 2.46 | 2.19 |
|  | IC524639 | 1.57 | 1.17 | 11.00 | 27.33 | 106 | 5.23 | 4.20 | 10.00 | 33 | 37 | 67 | 14.60 | 44.03 | 2.53 | 1.96 |
|  | IC553505 | 1.57 | 1.13 | 13.00 | 48.00 | 100 | 6.23 | 5.43 | 13.00 | 35 | 39 | 65 | 27.80 | 43.10 | 2.79 | 2.05 |
|  | IC553509 | 1.98 | 1.63 | 10.50 | 44.67 | 88 | 6.00 | 5.57 | 13.33 | 38 | 42 | 70 | 27.20 | 43.23 | 2.73 | 2.26 |
|  | IC553510 | 1.93 | 1.27 | 12.67 | 30.33 | 110 | 5.67 | 3.43 | 11.67 | 39 | 42 | 69 | 25.80 | 37.83 | 2.64 | 2.38 |
|  | IC553512 | 1.90 | 1.30 | 11.00 | 21.33 | 70 | 5.73 | 4.80 | 12.33 | 38 | 42 | 68 | 13.80 | 35.70 | 2.51 | 2.11 |
|  | IC553516 | 1.77 | 1.30 | 10.90 | 28.33 | 120 | 5.37 | 3.00 | 7.67 | 39 | 43 | 69 | 22.40 | 43.77 | 2.98 | 2.27 |
|  | IC553517 | 1.73 | 1.30 | 10.00 | 31.67 | 86 | 5.17 | 3.87 | 11.00 | 31 | 35 | 63 | 18.00 | 43.47 | 2.43 | 2.39 |
|  | IC553518 | 1.90 | 1.13 | 9.57 | 51.67 | 87 | 5.00 | 4.23 | 12.33 | 35 | 39 | 65 | 17.60 | 40.40 | 2.97 | 2.07 |
|  | IC553520 | 1.87 | 1.27 | 10.17 | 33.00 | 84 | 5.30 | 4.90 | 11.67 | 34 | 38 | 66 | 19.80 | 37.33 | 2.65 | 2.23 |
|  | IC553521 | 1.92 | 1.40 | 10.50 | 29.67 | 100 | 4.30 | 4.37 | 12.67 | 34 | 38 | 66 | 13.40 | 43.90 | 2.71 | 2.40 |
|  | IC553522 | 1.96 | 1.67 | 14.50 | 32.33 | 147 | 5.53 | 4.40 | 12.00 | 31 | 35 | 64 | 28.00 | 41.67 | 2.66 | 2.45 |
|  | IC553523 | 1.53 | 1.30 | 11.50 | 28.67 | 112 | 6.13 | 5.43 | 12.33 | 32 | 36 | 63 | 26.80 | 45.53 | 2.98 | 1.97 |
|  | IC553524 | 1.83 | 1.43 | 9.17 | 30.00 | 129 | 5.47 | 4.37 | 11.00 | 33 | 37 | 64 | 22.00 | 41.93 | 2.66 | 2.03 |
|  | IC553525 | 1.95 | 1.50 | 14.53 | 35.83 | 106 | 6.33 | 3.87 | 9.33 | 38 | 43 | 68 | 22.40 | 45.53 | 2.90 | 2.26 |
|  | IC553526 | 1.94 | 1.37 | 12.67 | 36.00 | 156 | 6.67 | 4.20 | 10.00 | 33 | 37 | 65 | 19.80 | 41.03 | 2.65 | 2.04 |
|  | IC553534 | 1.53 | 1.17 | 11.83 | 32.33 | 78 | 4.57 | 4.27 | 12.67 | 36 | 40 | 65 | 6.60 | 41.03 | 2.88 | 1.97 |
|  | IC553535 | 1.75 | 1.19 | 11.00 | 35.67 | 57 | 5.47 | 4.43 | 10.67 | 35 | 39 | 63 | 10.20 | 40.93 | 2.92 | 2.85 |
|  | IC553527 | 2.17 | 1.62 | 13.33 | 37.67 | 133 | 5.83 | 5.07 | 13.33 | 36 | 40 | 67 | 13.20 | 43.23 | 2.56 | 2.17 |
|  | IC553528 | 2.23 | 1.65 | 13.00 | 38.67 | 123 | 5.83 | 5.20 | 14.00 | 36 | 40 | 67 | 22.00 | 43.23 | 2.56 | 2.17 |
|  | IC553529 | 2.20 | 1.70 | 12.33 | 44.00 | 105 | 5.63 | 4.63 | 15.00 | 39 | 43 | 68 | 22.00 | 43.10 | 2.52 | 2.21 |
|  | IC553530 | 2.37 | 1.63 | 11.00 | 42.67 | 78 | 4.83 | 3.67 | 11.67 | 36 | 40 | 67 | 15.20 | 40.13 | 2.80 | 2.07 |
|  | IC553531 | 2.48 | 1.64 | 11.33 | 39.00 | 72 | 5.33 | 4.67 | 12.67 | 32 | 36 | 65 | 18.80 | 41.90 | 2.73 | 2.06 |
|  | IC553532 | 2.37 | 1.56 | 10.17 | 43.67 | 94 | 5.33 | 4.33 | 12.00 | 33 | 37 | 64 | 11.00 | 45.50 | 2.64 | 2.35 |
|  | IC553537 | 1.73 | 1.10 | 13.17 | 30.00 | 92 | 6.33 | 4.97 | 13.00 | 36 | 40 | 65 | 14.20 | 45.50 | 2.47 | 2.05 |
|  | IC553538 | 1.63 | 1.23 | 11.33 | 46.00 | 125 | 5.13 | 4.87 | 12.00 | 36 | 39 | 64 | 7.40 | 44.57 | 2.69 | 2.24 |
|  | IC553539 | 1.90 | 1.13 | 14.30 | 30.00 | 77 | 5.50 | 4.80 | 13.00 | 40 | 43 | 67 | 16.00 | 44.57 | 2.80 | 2.14 |
|  | IC553540 | 1.93 | 1.20 | 13.00 | 56.67 | 130 | 5.07 | 4.60 | 10.67 | 40 | 43 | 66 | 14.40 | 44.27 | 2.62 | 2.13 |
|  | IC553541 | 1.86 | 1.20 | 7.33 | 34.00 | 65 | 4.40 | 4.47 | 11.33 | 34 | 38 | 65 | 10.20 | 37.20 | 2.90 | 2.35 |
|  | IC553544 | 1.10 | 1.07 | 8.10 | 21.67 | 45 | 6.12 | 4.63 | 11.67 | 35 | 39 | 65 | 8.60 | 45.40 | 2.65 | 2.25 |
|  | IC553547 | 1.90 | 1.40 | 9.67 | 41.33 | 96 | 4.80 | 4.67 | 13.00 | 38 | 42 | 66 | 19.20 | 43.50 | 2.76 | 2.16 |
|  | IC553548 | 1.65 | 1.26 | 7.67 | 32.33 | 61 | 5.05 | 4.67 | 12.33 | 39 | 43 | 67 | 10.60 | 42.43 | 1.76 | 1.51 |
|  | IC553551 | 1.73 | 1.13 | 12.67 | 47.33 | 105 | 5.03 | 4.77 | 11.33 | 37 | 41 | 66 | 12.00 | 42.73 | 2.51 | 1.81 |
|  | IC550520 | 2.01 | 1.47 | 10.67 | 27.00 | 82 | 6.17 | 4.57 | 11.33 | 34 | 38 | 67 | 12.80 | 44.33 | 2.46 | 1.98 |
|  | IC553553 | 2.63 | 1.10 | 12.00 | 34.00 | 70 | 6.73 | 4.30 | 10.33 | 37 | 41 | 68 | 13.20 | 41.87 | 2.94 | 2.78 |
|  | IC553554 | 2.53 | 1.27 | 12.33 | 37.67 | 170 | 5.40 | 4.03 | 8.67 | 38 | 42 | 67 | 12.00 | 43.87 | 2.78 | 2.28 |
|  | IC553555 | 2.01 | 1.13 | 12.50 | 32.00 | 65 | 5.70 | 4.90 | 11.33 | 39 | 43 | 69 | 8.20 | 38.47 | 2.19 | 2.04 |
|  | IC553556 | 2.35 | 1.17 | 6.50 | 49.00 | 110 | 4.60 | 5.27 | 13.33 | 37 | 41 | 67 | 19.40 | 36.77 | 2.83 | 2.31 |
|  | IC553557 | 2.07 | 1.30 | 12.33 | 56.00 | 100 | 4.63 | 4.33 | 11.33 | 34 | 42 | 67 | 13.60 | 42.30 | 2.78 | 2.25 |
|  | IC553558 | 2.10 | 1.07 | 12.00 | 40.00 | 134 | 5.00 | 4.57 | 11.67 | 38 | 43 | 68 | 23.00 | 37.43 | 2.97 | 2.18 |
|  | IC553560 | 2.01 | 1.03 | 11.50 | 33.00 | 68 | 5.47 | 5.40 | 12.67 | 38 | 42 | 67 | 17.40 | 42.23 | 2.15 | 2.09 |
|  | IC553561 | 2.00 | 1.40 | 8.00 | 36.33 | 84 | 4.73 | 4.70 | 12.67 | 38 | 41 | 68 | 17.40 | 41.20 | 2.45 | 2.16 |
|  | IC553562 | 2.20 | 1.17 | 10.50 | 32.67 | 56 | 5.20 | 4.60 | 13.67 | 38 | 42 | 67 | 7.80 | 40.67 | 2.45 | 2.14 |
|  | IC553564 | 2.50 | 1.50 | 12.83 | 63.00 | 102 | 6.90 | 3.77 | 10.67 | 39 | 43 | 65 | 17.40 | 37.20 | 2.95 | 2.36 |
|  | IC553565 | 2.00 | 1.30 | 10.83 | 27.00 | 85 | 5.27 | 5.17 | 13.00 | 37 | 41 | 66 | 13.80 | 41.93 | 2.98 | 2.78 |
|  | IC622860 | 1.23 | 1.03 | 8.00 | 24.00 | 101 | 3.27 | 5.30 | 13.00 | 40 | 40 | 58 | 9.20 | 43.23 | 2.83 | 2.03 |
|  | IC622861 | 1.23 | 1.03 | 9.77 | 24.67 | 105 | 3.23 | 5.13 | 12.67 | 42 | 38 | 53 | 8.00 | 42.27 | 2.80 | 2.01 |
|  | IC276983 | 1.17 | 1.13 | 9.03 | 23.33 | 110 | 3.57 | 5.17 | 13.33 | 44 | 40 | 55 | 7.20 | 43.27 | 2.77 | 2.05 |
|  | IC622865 | 1.11 | 1.07 | 9.33 | 23.12 | 105 | 3.73 | 5.40 | 15.00 | 40 | 44 | 56 | 5.00 | 36.27 | 2.64 | 2.07 |
|  | NS_1 | 1.10 | 1.03 | 8.17 | 23.15 | 107 | 3.27 | 5.20 | 12.67 | 42 | 47 | 50 | 8.40 | 35.90 | 2.58 | 2.02 |
|  | IC251435 | 1.27 | 1.03 | 10.67 | 32.67 | 144 | 4.75 | 4.57 | 11.00 | 34 | 38 | 63 | 13.00 | 44.83 | 2.63 | 1.86 |
|  | BD_1 | 1.33 | 1.07 | 12.00 | 31.15 | 141 | 4.71 | 4.10 | 10.00 | 35 | 39 | 64 | 13.00 | 45.17 | 2.63 | 1.84 |
|  | IC251436 | 1.13 | 1.00 | 3.50 | 27.33 | 82 | 3.53 | 4.47 | 12.33 | 21 | 24 | 51 | 5.80 | 37.00 | 2.65 | 1.81 |
|  | IC331436 | 1.10 | 0.90 | 3.83 | 25.67 | 75 | 3.52 | 4.10 | 11.67 | 22 | 25 | 52 | 6.80 | 38.70 | 2.62 | 1.84 |
|  | IC331437 | 1.10 | 1.00 | 4.33 | 26.67 | 72 | 3.56 | 4.47 | 12.00 | 20 | 23 | 49 | 6.20 | 41.67 | 2.66 | 2.22 |
|  | IC331453 | 1.33 | 0.93 | 15.17 | 42.00 | 140 | 3.81 | 3.00 | 13.00 | 60 | 64 | 66 | 8.20 | 41.23 | 2.93 | 2.37 |
|  | IC331454 | 1.57 | 1.10 | 14.87 | 41.30 | 145 | 3.85 | 3.87 | 11.00 | 56 | 59 | 59 | 4.60 | 41.50 | 2.34 | 2.08 |
|  | IC331456 | 1.60 | 1.33 | 17.20 | 40.21 | 148 | 3.97 | 4.23 | 13.00 | 58 | 62 | 62 | 5.20 | 43.27 | 2.76 | 1.62 |
|  | IC331457 | 1.40 | 1.10 | 17.67 | 41.30 | 152 | 3.78 | 4.90 | 12.67 | 59 | 63 | 59 | 6.20 | 41.60 | 2.57 | 1.95 |
|  | IC331610 | 1.50 | 1.07 | 16.31 | 41.33 | 132 | 3.95 | 4.37 | 13.33 | 62 | 66 | 61 | 8.40 | 41.90 | 2.85 | 2.15 |
|  | IC251438 | 1.47 | 1.07 | 8.83 | 32.67 | 95 | 2.96 | 4.70 | 12.33 | 40 | 45 | 76 | 6.60 | 41.43 | 2.57 | 1.55 |
|  | IC349701 | 1.40 | 1.30 | 6.33 | 23.33 | 112 | 2.67 | 4.40 | 12.67 | 35 | 39 | 64 | 4.00 | 40.20 | 2.25 | 1.98 |
|  | IC351406 | 1.50 | 1.20 | 5.37 | 19.00 | 98 | 2.80 | 5.43 | 13.67 | 40 | 44 | 67 | 5.60 | 46.03 | 2.57 | 2.05 |
|  | IC417392 | 1.27 | 1.01 | 7.83 | 32.00 | 116 | 3.80 | 3.87 | 9.67 | 43 | 48 | 73 | 5.80 | 40.07 | 2.51 | 2.31 |
|  | IC622867 | 1.53 | 1.10 | 9.10 | 35.33 | 125 | 3.53 | 4.97 | 15.00 | 32 | 35 | 57 | 5.60 | 44.10 | 2.16 | 1.47 |
|  | IC622868 | 1.37 | 1.13 | 7.33 | 25.00 | 110 | 3.87 | 3.70 | 11.67 | 33 | 37 | 61 | 5.00 | 43.27 | 2.66 | 2.22 |
|  | IC622869 | 1.53 | 1.30 | 6.00 | 25.33 | 100 | 2.83 | 4.67 | 12.67 | 38 | 42 | 72 | 4.54 | 42.27 | 2.93 | 2.37 |
|  | IC521211 | 1.13 | 1.20 | 7.70 | 17.67 | 151 | 3.10 | 4.63 | 12.67 | 39 | 43 | 66 | 6.25 | 37.03 | 2.46 | 1.94 |
|  | IC521245 | 1.10 | 1.23 | 9.00 | 22.00 | 98 | 3.25 | 4.97 | 13.67 | 38 | 41 | 67 | 5.80 | 37.97 | 2.66 | 2.22 |
|  | IC521215 | 1.15 | 1.23 | 9.00 | 22.00 | 98 | 3.03 | 4.97 | 13.67 | 38 | 41 | 67 | 5.80 | 37.97 | 2.66 | 2.22 |
|  | Pk_101 | 1.17 | 1.13 | 7.50 | 20.00 | 65 | 3.17 | 4.37 | 10.33 | 37 | 41 | 65 | 7.35 | 35.57 | 2.93 | 2.37 |
|  | IC37804 | 1.10 | 1.17 | 9.10 | 48.00 | 91 | 3.85 | 4.77 | 12.33 | 39 | 43 | 68 | 7.00 | 36.40 | 3.17 | 2.34 |
|  | IC622870 | 1.63 | 1.10 | 4.50 | 11.00 | 70 | 2.13 | 4.43 | 13.00 | 37 | 41 | 65 | 5.60 | 44.50 | 3.13 | 2.06 |
|  | BDD_1 | 1.51 | 1.10 | 4.50 | 11.00 | 96 | 2.13 | 4.43 | 13.00 | 37 | 41 | 65 | 5.60 | 44.50 | 3.13 | 2.06 |
|  | IC421767 | 2.32 | 1.07 | 6.33 | 28.00 | 72 | 4.41 | 4.23 | 13.00 | 28 | 32 | 73 | 4.78 | 37.30 | 2.84 | 2.00 |
|  | IC24830 | 2.10 | 1.10 | 6.00 | 26.12 | 68 | 4.30 | 4.30 | 12.33 | 26 | 31 | 75 | 5.21 | 44.87 | 2.63 | 1.86 |
|  | NV_1 | 1.11 | 0.96 | 4.87 | 21.33 | 101 | 4.01 | 4.80 | 13.00 | 45 | 49 | 67 | 12.40 | 41.67 | 2.65 | 1.81 |
|  | IC625694 | 1.21 | 0.99 | 5.12 | 22.15 | 106 | 4.12 | 4.27 | 13.00 | 46 | 51 | 68 | 4.40 | 41.27 | 2.48 | 1.90 |
