# Supplementary material for: Insights into the genetic diversity of an underutilized Indian legume, Vigna stipulacea (Lam.) Kuntz., using morphological traits and microsatellite markers
Source: PLoS One. 2022 Jan 19;17(1):e0262634. doi: 10.1371/journal.pone.0262634 (PMC8769370; doi:10.1371/journal.pone.0262634)
Supplement: S4 Table — (DOCX) [file pone.0262634.s004.docx]

# S4 Table. Mean value inferred from model-based approach.

| Mean value of alpha | 0.0340 |
| --- | --- |
| Mean value of Fst_1 | 0.3362 |
| Mean value of Fst_2 | 0.4473 |
| Mean value of Fst_3 | 0.6385 |
| Mean value of Fst_4 | 0.6902 |
| Mean value of Fst_5 | 0.5324 |
