# Supplementary material for: Insights into the genetic diversity of an underutilized Indian legume, Vigna stipulacea (Lam.) Kuntz., using morphological traits and microsatellite markers
Source: PLoS One. 2022 Jan 19;17(1):e0262634. doi: 10.1371/journal.pone.0262634 (PMC8769370; doi:10.1371/journal.pone.0262634)
Supplement: S5 Table — (DOCX) [file pone.0262634.s005.docx]

# S5 Table. Analysis of molecular variance of 33 SSRs among 94 *V. stipulacea* accessions

| **Source** | **df** | **SS** | **MS** | **Est. Var.** | **%** |
| --- | --- | --- | --- | --- | --- |
| **Among Populations** | 4 | 506.158 | 126.540 | 3.155 | 39% |
| **Among Individuals** | 89 | 833.714 | 9.368 | 4.426 | 55% |
| **Within Individuals** | 94 | 48.500 | 0.516 | 0.516 | 6% |
| **Total** | 187 | 1388.372 |  | 8.097 | 100% |
