# Supplementary figures and images for: Insights into the genetic diversity of an underutilized Indian legume, Vigna stipulacea (Lam.) Kuntz., using morphological traits and microsatellite markers
Source: PLoS One. 2022 Jan 19;17(1):e0262634. doi: 10.1371/journal.pone.0262634 (PMC8769370; doi:10.1371/journal.pone.0262634)

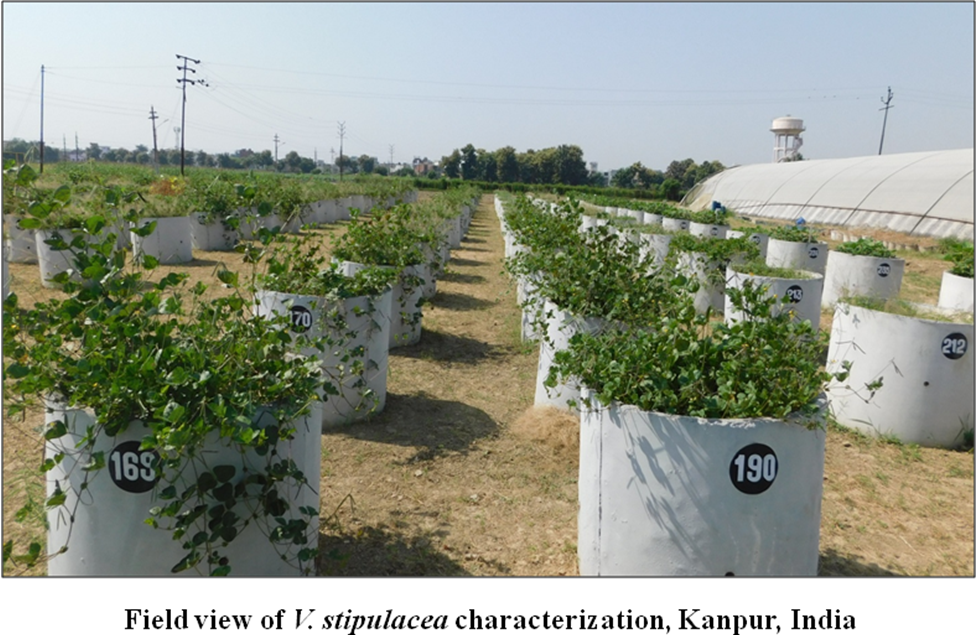

Supplement: S1 Fig — (TIF) [file pone.0262634.s006.tif]

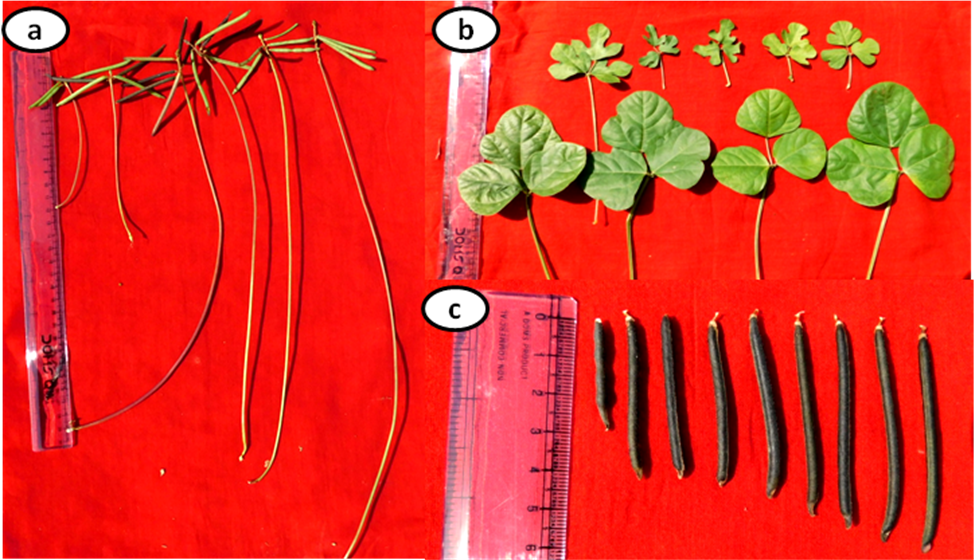

Supplement: S2 Fig — Variability in morphological traits viz. a) peduncle length; b) leaf shape and size; c) pod length. (TIF) [file pone.0262634.s007.tif]

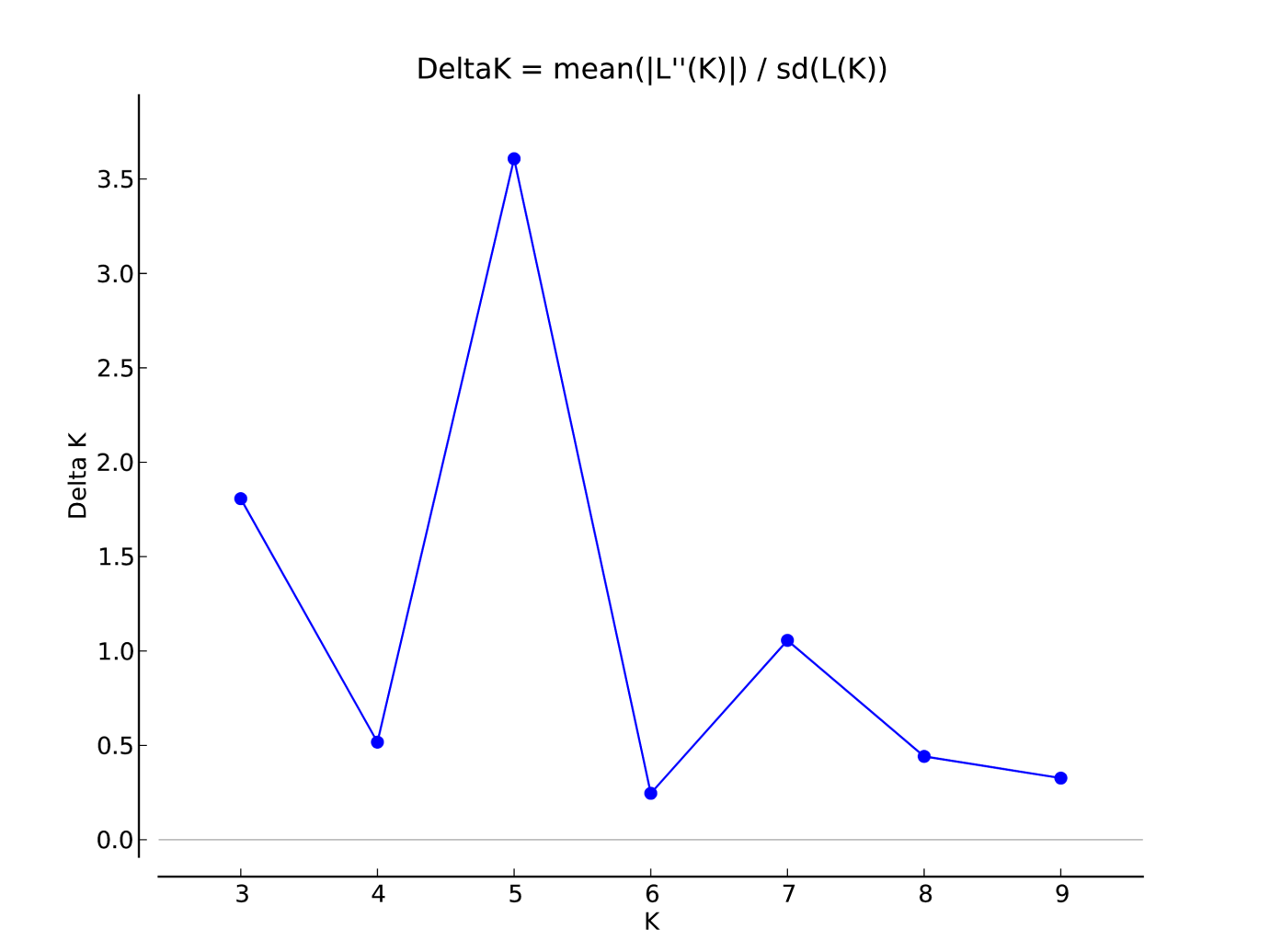

Supplement: S3 Fig — (TIF) [file pone.0262634.s008.tif]
